# Developmental Changes in Task‐Induced Brain Deactivation in Humans Revealed by a Motor Task

**DOI:** 10.1002/dneu.22701

**Published:** 2019-06-10

**Authors:** Tomoyo Morita, Minoru Asada, Eiichi Naito

**Affiliations:** ^1^ Graduate School of Engineering Osaka University 2‐1 Yamadaoka Suita Osaka 565‐0871 Japan; ^2^ Center for Information and Neural Networks (CiNet) National Institute of Information and Communications Technology (NICT) 2A6 1‐4 Yamadaoka Suita Osaka 565‐0871 Japan; ^3^ Graduate School of Frontier Biosciences Osaka University 1‐1 Yamadaoka Suita Osaka 565‐0871 Japan

**Keywords:** brain deactivation, cross‐modal inhibition, development, functional MRI, ipsilateral sensorimotor cortex

## Abstract

Performing tasks activates relevant brain regions in adults while deactivating task‐irrelevant regions. Here, using a well‐controlled motor task, we explored how deactivation is shaped during typical human development and whether deactivation is related to task performance. Healthy right‐handed children (8–11 years), adolescents (12–15 years), and young adults (20–24 years; 20 per group) underwent functional magnetic resonance imaging with their eyes closed while performing a repetitive button‐press task with their right index finger in synchronization with a 1‐Hz sound. Deactivation in the ipsilateral sensorimotor cortex (SM1), bilateral visual and auditory (cross‐modal) areas, and bilateral default mode network (DMN) progressed with development. Specifically, ipsilateral SM1 and lateral occipital deactivation progressed prominently between childhood and adolescence, while medial occipital (including primary visual) and DMN deactivation progressed from adolescence to adulthood. In adults, greater cross‐modal deactivation in the bilateral primary visual cortices was associated with higher button‐press timing accuracy relative to the sound. The region‐specific deactivation progression in a developmental period may underlie the gradual promotion of sensorimotor function segregation required in the task. Task‐induced deactivation might have physiological significance regarding suppressed activity in task‐irrelevant regions. Furthermore, cross‐modal deactivation develops to benefit some aspects of task performance in adults.

## Introduction

Many neuroimaging studies have shown that performing a task activates relevant brain regions, while it may deactivate task‐irrelevant regions in the adult brain. Deactivation usually refers to the observation that brain activity decreases in an experimental condition (task) compared to that in another (control) condition. Such task‐induced deactivation has been reported in both early positron emission tomography studies (manifesting as a decrease in regional cerebral blood flow; rCBF; Haxby *et al.*, 1994; Kawashima *et al.*, 1995; Sadato *et al.*, 1996; 1998; Shulman *et al.*, 1997) and more recent functional magnetic resonance imaging (fMRI) studies (Lewis *et al.*, 2000; Laurienti *et al.*, 2002; McKiernan *et al.*, 2003; Amedi *et al.*, 2005; Weisser *et al.*, 2005; McKiernan *et al.*, 2006; Jorge *et al.*, 2018). In the latter, deactivation is called as negative blood oxygenation level‐dependent (BOLD) phenomenon. Although its physiological mechanisms are not fully understood (Kim and Ogawa, 2012; Moraschi *et al.*, 2012), accumulating evidence suggests that the neuronal suppression (inhibition) is an important contributor to task‐induced negative BOLD signals (Shmuel *et al.*, 2002; 2006; Smith *et al.*, 2004; Boorman *et al.*, 2010; Wade and Rowland, 2010; Millinger *et al*., 2014; Sten *et al*., 2017).

In the neuroimaging research conducted in adults, task‐induced deactivation phenomena (decreased rCBF and negative BOLD) have been well documented in sensory (visual, auditory, and tactile) tasks (cross‐modal inhibition; see the references above). Similarly, deactivation during motor tasks has also been reported in the adult ipsilateral primary sensorimotor cortex (SM1; Allison *et al.*, 2000; Newton *et al.*, 2005; Marchand *et al.*, 2007; Hayashi *et al.*, 2008), visual and auditory cross‐modal areas (Jäncke *et al.*, 2000), and the default mode network (DMN; Marchand *et al.*, 2007). These lines of evidence seem to suggest that task‐induced deactivation may occur in brain regions irrelevant to performing a task in a manner that suggests physiological significance and benefits task performance. If deactivation is somehow associated with neuronal suppression in the brain regions irrelevant to performing a task, deactivation patterns may change with development alongside the progression of functional segregation among distributed brain networks. However, these developmental dynamics have never been systematically described. In addition, it is not fully understood whether deactivation benefits task performing.

In the present study, we used a motor task and explored how the task shapes the deactivation pattern in the entire brain and how the patterns change during typical human development. We used fMRI to examine regional brain activity in healthy children, adolescents, and young adults performing a well‐controlled repetitive button‐press task with their right index finger in synchronization with a 1‐Hz sound; this task was a finger tapping task, which is representative of a paradigm used in a neuroimaging investigation of the human motor system (Witt *et al.*, 2008). Based on previous studies in adults, we expected to detect deactivation of the ipsilateral SM1, cross‐modal areas, and DMN. We report the developmental changes in deactivation in the entire brain, as well as developmental changes in brain activation associated with the motor task. As for the performance of the motor task, we computed the absolute time difference between each 1‐Hz sound and its corresponding button‐press (absolute asynchrony) and any variance of asynchrony; we also examined whether deactivation is related to these behavioral indexes that reflect task performance.

## Materials and Methods

### Participants

Twenty healthy right‐handed children (CH; 9 boys and 11 girls; mean age: 9.7 ± 0.9 years; range: 8 years 7 months–11 years 9 months), 20 adolescents (ADO; 10 boys and 10 girls; mean age: 13.9 ± 0.9 years; range: 12 years 5 months–15 years 2 months), and 20 young adults (AD; 11 males and 9 females; mean age: 21.6 ± 1.1 years; range: 20 years 0 months–24 years 0 months) participated in this study. The children, adolescents, and adults were recruited from local elementary and junior high schools as well as universities. We also collected data from another three children and one adolescent; however, we excluded their data from the analysis because of their excessive head motions (more than 3 mm on either the *x*, *y*, or *z* axis) during fMRI scanning, according to a previous study (Morita *et al.*, 2018). We confirmed handedness with the Edinburgh Handedness Inventory (Oldfield, 1971) and ensured that no participants had a history of neurological or psychiatric disorders based on self‐reports and those provided by legal guardians.

The study protocol was approved by the ethics committee of the National Institute of Information and Communications Technology. We explained the details of the study to the participants before the start of the experiment, after which the participants provided written informed consent. For the children and adolescents, we also obtained written informed consent from their legal guardians. The study was carried out following the principles and guidelines of the Declaration of Helsinki (1975).

### Button‐Press Task

Before the fMRI experiment, the participants familiarized themselves with a button‐press task by performing it outside the scanner, before they entered the MR room. The participants then laid in the fMRI scanner with their ears plugged. We fastened their heads to the head coil with adhesive tape and immobilized them with sponge cushions to reduce possible head motions. Both arms of the participants were naturally semipronated and extended in front of them. We asked the participants to relax their entire bodies without producing unnecessary movements and to think only of things relevant to the tasks assigned.

The participants were asked to press an MR‐compatible button (Current Design Inc., Philadelphia, PA) with their right index finger precisely in synchronization with a 1‐Hz sound generated by a computer. Each participant completed two experimental runs, each lasting 160 s. Each run was composed of five button‐press epochs (Button‐press) of 15 s each. The button‐press epochs were separated by resting periods of 15 s (Baseline). During the resting period (Rest), a 1‐Hz sound at a different pitch was produced. Thus, during the Rest period, participants received auditory stimuli at a rate of 1‐Hz but did not move their fingers. Each run also included a 15‐s period before the start of the first button‐press epoch. Throughout an fMRI run, the participants kept their eyes closed and their right index fingers on the button, and performed repetitive button‐press without releasing the finger from the button. We asked the participants to close their eyes just before we started an fMRI run because eye closure duration greatly affects activity in visual areas (Weisser *et al.*, 2005; Merabet *et al.*, 2007). We confirmed that the participants kept their eyes closed and performed 1‐Hz repetitive button‐press without releasing their fingers from the button by visual inspection throughout each run.

We adopted this simple motor task because it allowed us to control the strategies and contents of motor behaviors across participants and could be successfully performed by all participants (Lewis *et al.*, 2004). During the fMRI run, we gave the participants auditory instructions (“3, 2, 1, start” and “stop”) through MR‐compatible headphones to inform them of the start and finish of a button‐press epoch. These instructions were also generated by a computer.

**Figure 1:**
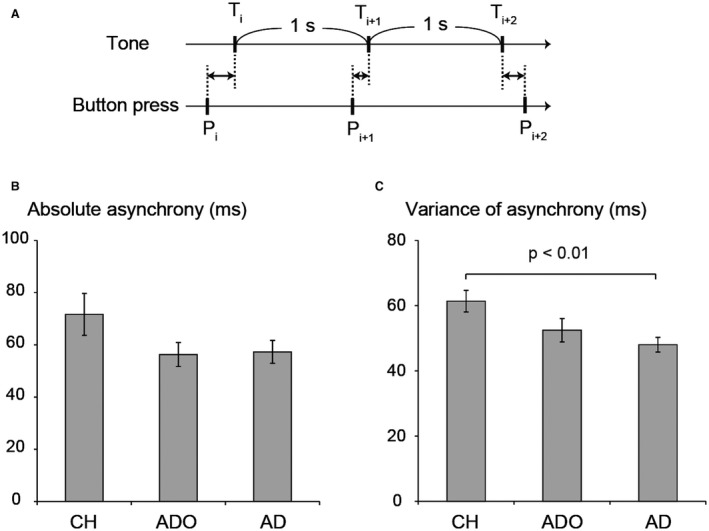
The performance of children (CH), adolescents (ADO), and adults (AD) in the auditory synchronized button‐press task. (A) The participants pressed a magnetic resonance (MR)‐compatible button with their right index fingers in synchronization with a 1‐Hz sound with their eyes closed. We recorded the timings of the individual tones (*T_i_*, *T_i_*_ + 1_, *T_i_*_ + 2_…) and corresponding button‐press (*P_i_*, *P_i_*_ + 1_, *P_i_*_ + 2_…) for every participant, and computed the absolute time difference between each tone of the 1‐Hz sound and the corresponding button‐press (|*T_i_* –*P_i_*|; absolute asynchrony). We also computed the standard deviation of within‐epoch asynchrony (variance of asynchrony). These were calculated in each epoch for every participant, which were used in the following parametric modulation analysis. We also calculated the average absolute asynchrony and the variance of asynchrony per epoch across the two experimental runs and used them as a measure of performance of each participant. (B) The absolute asynchrony and (C) variance of asynchrony in the three age groups. The absolute asynchrony tended to be greater in the CH group than in the other groups; however, no significant differences were found among groups. The variance of asynchrony in the CH group was significantly greater than that in the AD group.

The timings of the 1‐Hz sounds and button‐press for each participant were computer recorded. In the analysis, we counted the number of button‐press responses in each button‐press epoch, and calculated the average number of button‐press responses per epoch across the two experimental runs for each participant. As for the task performance (Fig. 1A), in order to evaluate the accuracy of button‐press timing relative to the 1‐Hz sound, we computed the absolute time difference between each tone of the 1‐Hz sound and the corresponding button‐press (absolute asynchrony, Pecenka and Keller, 2011). In order to evaluate the variability of button‐press timing, we computed the standard deviation of within‐epoch asynchrony (variance of asynchrony; Pecenka and Keller, 2011). These indexes were calculated in each epoch for every participant and were used in the parametric modulation analysis that followed. In these calculations, we excluded data from the first and last button‐press in each epoch. We also calculated the average absolute asynchrony and variance of asynchrony per epoch across the two experimental runs for each participant. Statistical evaluation of the behavioral indexes was performed by one‐way analysis of variance (ANOVA; groups: CH, ADO, and AD) to evaluate the group differences.

### fMRI Data Acquisition

Functional images were acquired using T2*‐weighted gradient echo‐planar imaging (EPI) sequences with a 3.0‐Tesla MRI scanner (Trio Tim; SIEMENS, Germany) and a 32‐channel array coil. Each volume consisted of 44 slices (slice thickness = 3.0 mm, inter‐slice thickness = 0.5 mm) acquired in an ascending order, covering the entire brain. The time interval between successive acquisitions from the same slice was 2,500 ms. An echo time of 30 ms and a flip angle of 80 degrees were used. The field of view was 192 × 192 mm and the matrix size was 64 × 64. Voxel dimensions were 3 × 3 × 3.5 mm in the *x‐*, *y‐*, and *z*‐axes, respectively. We collected 64 volumes in each experimental run.

### Imaging Data Preprocessing

To eliminate the effects of unsteady magnetization during the task, we discarded the first four EPI images in each fMRI run before the first epoch started. Imaging data were analyzed using SPM 8 (Wellcome Trust Centre for Neuroimaging, London, UK) implemented in Matlab (Mathworks, Sherborn, MA).

EPI images were realigned to the first image and then to the mean image. Through this realignment procedure, we obtained head position data that changed over time from the first frame through six parameters (translational displacements along *x*‐, *y*‐, and *z*‐axes and the rotational displacements of pitch, raw, and roll). Then, we calculated the absolute value of displacement in each frame from its previous frame (framewise displacement [FD]; Power *et al.*, 2012). This was done for every translational and rotational axis. We totaled these values per frame. In this calculation, we converted rotational displacements from degrees to millimeters by calculating the displacement on the surface of a sphere with a radius 50 mm, which is approximately the mean distance from the cerebral cortex to the center of the head (Power *et al.*, 2012).

In order to check the change in FD values through all frames of an entire experimental run, we counted the number of frames that had an FD over 0.9 mm in each participant, according to a previous study (Siegel *et al.*, 2014). The number of frames in which FD exceeded 0.9 mm was 0 in the majority of participants (18/20, 18/20, 19/20 of the CH, ADO, and AD groups, respectively). Even in the participants who had such frames, the percentage was less than 5% of all 120 frames. The average FD of all frames across all participants was 0.087 ± 0.041 mm, 0.062 ± 0.031 mm, and 0.061 ± 0.026 mm for the CH, ADO, and AD groups, respectively. These values were much smaller when compared to those reported in a previous study (Engelhardt *et al.*, 2017), perhaps owing to our special care to prevent head motions. Although a one‐way ANOVA showed a significant group effect [*F* (2, 57) = 3.84, *p* < 0.05], post‐hoc Bonferroni *t*‐tests showed no significant group differences in any combinations. The FD during the button‐press epoch was smaller than that during the resting period, regardless of the age group, as shown in a previous cognitive study (Engelhardt *et al.*, 2017).

The realigned images were normalized to the Montreal Neurological Institute (MNI) space (Evans *et al.*, 1994). By comparing functional activation foci in children and adults within a common stereotaxic space, Kang *et al.* (2003) provided an empirical validation of MNI normalization for the analysis of fMRI data obtained from school‐aged children. Finally, the spatially normalized functional images were filtered using a Gaussian kernel with a full width at half maximum of 4 mm along the *x*‐, *y*‐, and *z*‐axes.

### Analysis of Button‐Press‐Related Deactivation/Activation Within Groups

After preprocessing, we first explored button‐press‐related deactivation, as well as activation, in each participant with a general linear model (GLM; Friston *et al.*, 1995; Worsley and Friston, 1995). The design matrix contained a boxcar function for the button‐press epoch that was convolved with a canonical hemodynamic response function. To correct for residual motion‐related variance after realignment, the six realignment parameters were also included in the design matrix as regressors of no interest.

We first generated a contrast image to examine brain regions that showed button‐press‐related deactivation (Rest > Button‐press) in each participant (single‐subject analyses). In this contrast image, the effects of the 1‐Hz sounds should be eliminated because the participants heard the sounds consistently in both the Button‐press epochs and Rest periods (see more in discussion). We also examined brain regions that showed button‐press‐related activation (Button‐press > Rest) in each participant. The contrast images from all participants were entered into a second‐level random effects group analysis (Holmes and Friston, 1998). One sample *t*‐tests were conducted for Rest > Button‐press (deactivation) and Button‐press > Rest (activation) in each group separately. In the second‐level analyses, we generated a voxel‐cluster image using an uncorrected voxel‐wise threshold of *p < *0.005 in each group. For statistical inference, we used a false discovery rate (FDR)‐corrected cluster‐wise threshold of *p* < 0.05 in the entire brain space (Genovese *et al.*, 2002).

To identify the anatomical regions corresponding to the deactivation/activation peaks, we referred to the cytoarchitectonic probability maps of the MNI standard brain in the SPM Anatomy Toolbox v2.2b (Eickhoff *et al.*, 2005). Functions that were probability assigned to each deactivation/activation region (peak) were verified in a publicly available database (http://www.neurosynth.org/locations/).

### Consistent Brain Deactivation/Activation Across Groups

**Figure 2:**
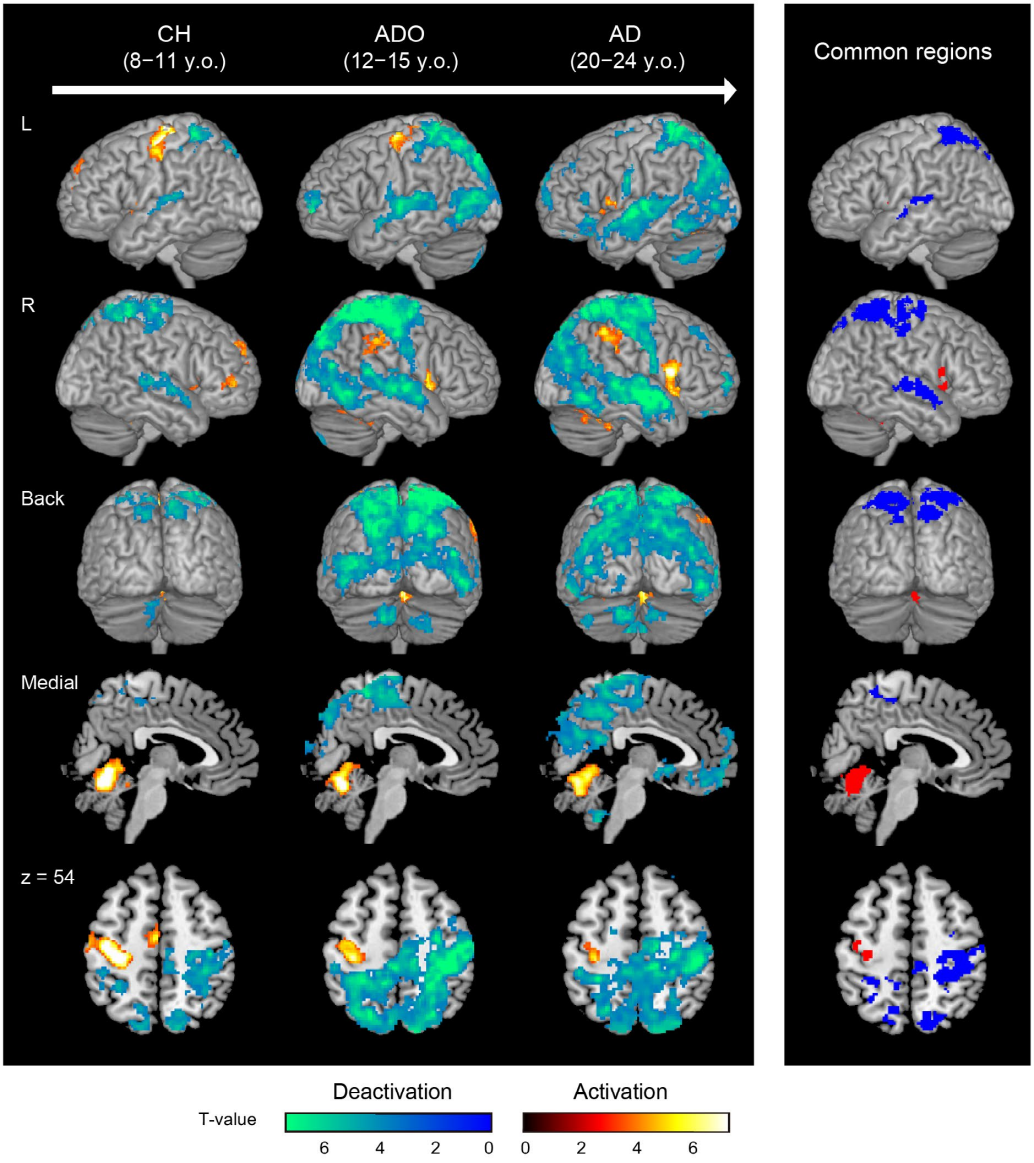
Button‐press‐related deactivation (cold color) and activation (warm color) in each group, and consistent deactivation (blue) and activation (red) across the groups. Deactivation and activation were rendered onto an MNI standard brain. The left hemisphere and right hemisphere, a back view (from top to bottom), a medial view (*x* = +4), and a horizontal section (*z* = +54) are shown in each column. The color bars at the bottom indicate *T*‐values. For display purposes, we showed left SM1 activations (42 and 35 voxels), though these clusters did not reach a significant level in the conjunction analysis. Abbreviations: AD, adults; ADO, adolescents; CH, children; L, left hemisphere; MNI, Montreal Neurological Institute; R, right hemisphere. [Colour figure can be viewed at wileyonlinelibrary.com]

To explore brain regions that consistently showed button‐press‐related deactivation (Rest > Button‐press) or activation (Button‐press > Rest) across all groups (Fig. 2), we performed conjunction analyses (Price and Friston, 1997). In these analyses, we generated a voxel‐cluster image using an uncorrected voxel‐wise threshold of *p < *0.005 and used an FDR‐corrected cluster‐wise threshold of *p* < 0.05 in the entire brain space.

### Comparisons Among Groups

**Figure 3:**
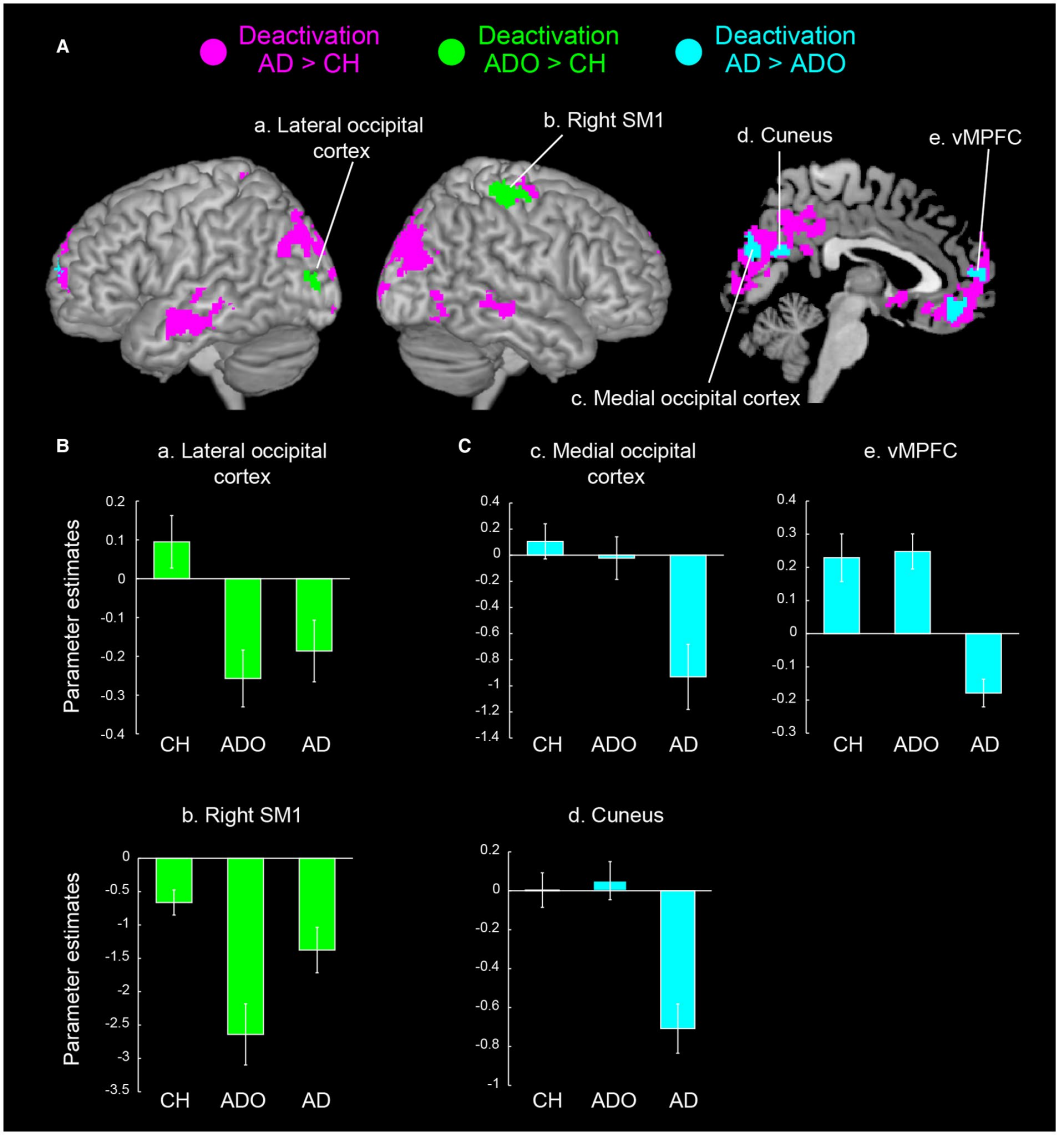
Group differences in button‐press‐related deactivation and their effect sizes (parameter estimates) in each group. (A) Brain regions in which deactivation significantly progressed in the AD group compared with that in the CH group (magenta), in the ADO group compared with that in the CH group (green), and in the AD group compared with that in the ADO group (light blue). Deactivation regions were rendered onto the left and right hemispheres, and the *x* = +2 sagittal section of an MNI standard brain. (B) The mean effect size in each group obtained from the green regions. The mean effect sizes obtained from the left lateral occipital cortex (peak coordinates = −16, −90, 8; region a in panel A) and the ipsilateral (right) SM1 (42, −34, 66; region b) are shown. The error bars indicate the standard error of the mean. (C) The mean effect size in each group obtained from the light blue regions. The mean effect sizes obtained from the medial occipital cortex (0, −80, 22; region c), cuneus (20, −62, 20; region d), and vMPFC (−12, 58, 16; region e) are shown. Abbreviations: AD, adults; ADO, adolescents; CH, children; MNI, Montreal Neurological Institute; SM1, primary sensorimotor cortex; vMPFC, ventro‐medial prefrontal cortex. [Colour figure can be viewed at wileyonlinelibrary.com]

To evaluate possible group differences, we first performed a one‐way ANOVA (groups: CH, ADO, and AD). We used the voxel‐wise threshold of *p* < 0.005 [*F* (2, 57) > 5.82]. In order to further examine whether the brain regions that showed group differences progress their deactivations in a particular developmental period (Fig. 3), we performed all possible comparisons among the three groups. For example, when we compared deactivation in the ADO group to that in the CH group [(Rest > Button‐press)_ADO_ – (Rest > Button‐press)_CH_], we used the image of (Rest > Button‐press)_ADO_ (uncorrected height threshold of *p* < 0.01) as an inclusive mask and the image of (Button‐press > Rest)_CH_ (uncorrected height threshold of *p* < 0.01) as an exclusive mask. Using these masking procedures, we ensured that any button‐press‐related deactivation that was greater in the ADO group was true deactivation rather than being caused by button‐press‐related activation in the CH group. We used the same procedure for the other deactivation comparisons. The validity of the masking procedure is discussed in our previous papers (Naito *et al.*, 2017; Morita *et al.*, 2018).

We also examined whether the brain regions that showed group differences progress their activations in a particular developmental period, by performing all possible comparisons among the three groups. For example, when we comparing activation in the CH group to that in the ADO group [(Button‐press > Rest)_CH_ – (Button‐press > Rest)_ADO_], we used the image of (Button‐press > Rest)_CH_ (uncorrected height threshold of *p* < 0.01) as an inclusive mask and the image of (Rest > Button‐press)_ADO_ (uncorrected height threshold of *p* < 0.01) as an exclusive mask. Using these masking procedures, we ensured that any button‐press‐related activation that was greater in the CH group was true activation rather being caused by button‐press‐related deactivation in the ADO group. We used the same procedure for the other activation comparisons.

In these group comparisons, we used an FDR‐corrected extent threshold of *p* < 0.05 in the entire brain for a voxel‐cluster image generated with a cluster‐defining height threshold of *p* < 0.005 uncorrected in each comparison.

To visualize the effect size in each identified brain region, we displayed the mean parameter estimate across the participants in each group. We extracted parameter estimates in each participant from the strongest peak of each voxel‐cluster obtained from the group analysis. We then calculated the mean parameter estimate across the participants in each group. Such visualization ensured the validity of our findings in the between‐group comparisons (see also Naito *et al.*, 2017). Hence, we did not perform any statistical evaluations of the parameter estimation data, thus avoiding the circular evaluation issue raised by Kriegeskorte *et al.* (2009).

### Correlation Analysis (Related to Task Performance)

Finally, we examined whether brain deactivation was related to task performance. In the behavioral analyses (see above), we calculated both the absolute asynchrony and the variance of asynchrony in each epoch for each participant (Fig. 1A). We used these values as behavioral indexes of task performance. Next, we performed a parametric modulation analysis (Büchel *et al.*, 1998), in which we constructed a second GLM by adding regressors of the behavioral indexes to the first GLM to test whether the degree of deactivation in a brain region covaried with epoch‐by‐epoch fluctuations of the absolute asynchrony and variance of asynchrony. The design matrix for this GLM contained a regressor for the Button‐press epoch and its corresponding linear regressors for the absolute asynchrony and variance of asynchrony.

**Figure 4:**
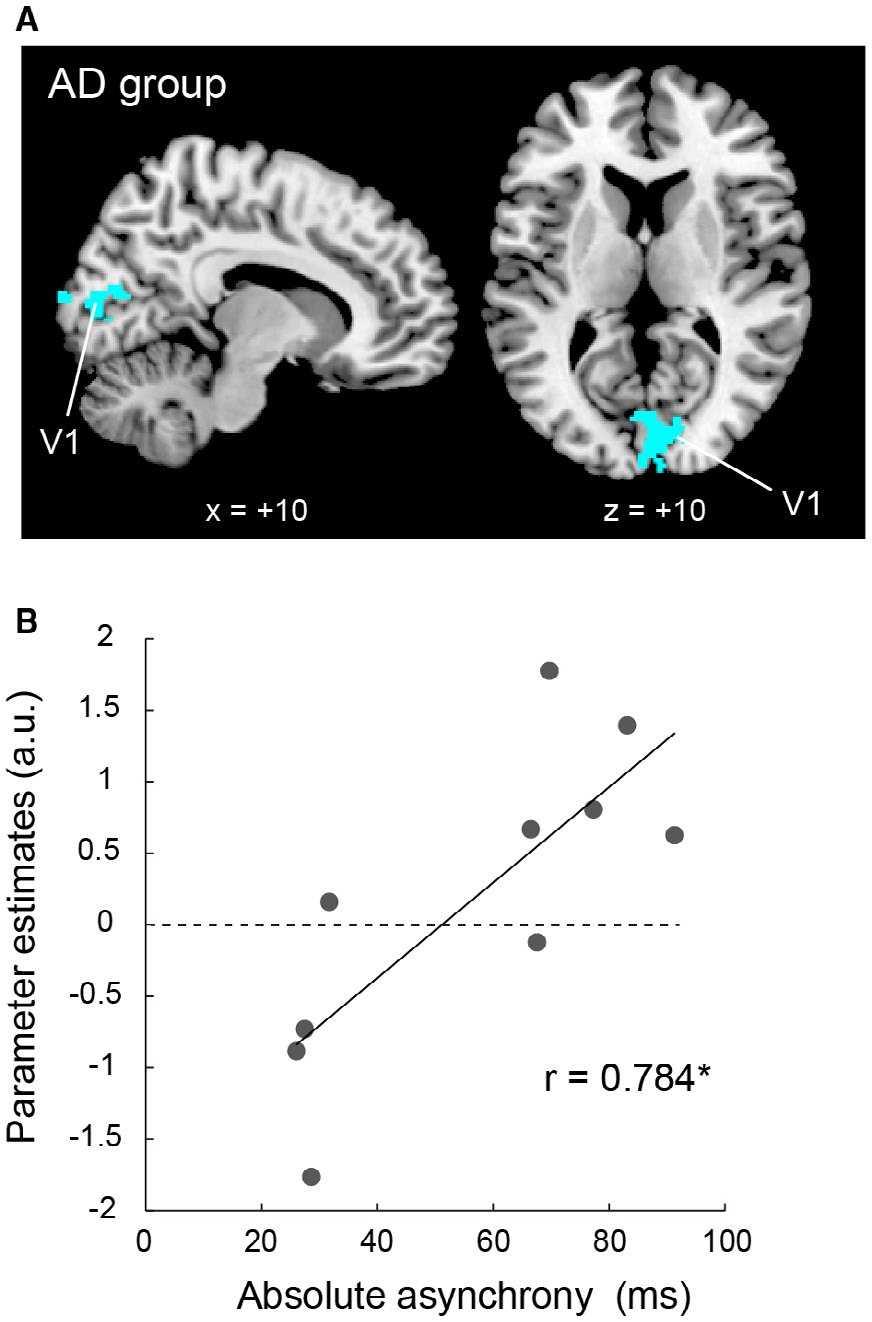
The correlation between deactivation and task performance. (A) Visual areas in which deactivation was correlated with better task performance (smaller absolute asynchrony). These regions (light blue) are shown in sagittal (*x* = +10) and horizontal (*z* = +10) sections of the MNI standard brain. Such brain regions were only observed in the AD group. (B) An example of a representative participant demonstrating the relationship between absolute asynchrony and activity in a V1 voxel (10, −84, 10) identified as the peak voxel in the group analysis. The absolute asynchrony became smaller in an epoch in which the V1 was deactivated (*N* = 10, *r* = 0.784, *p < *0.05). Each dot represents the mean data obtained from each epoch of the two experimental runs. The vertical axis indicates parameter estimates (arbitrary unit; a.u.), and the horizontal axis indicates mean within‐epoch absolute asynchrony (ms). The solid line indicates a regression line fitted to the data. Abbreviations: AD, adults; MNI, Montreal Neurological Institute; V1, primary visual cortex. [Colour figure can be viewed at wileyonlinelibrary.com]

We explored the brain regions in which deactivation was correlated with each index in each participant (single‐subject analyses), and then entered these individual images into a second‐level random effects group analysis. This procedure was performed separately in each group. In this analysis, we used the image of (Button‐press > Rest), obtained with an uncorrected height threshold of *p* < 0.01, as an exclusive mask. Using this masking procedure, we ensured that deactivation, rather than activation, was correlated with each index. In each group, we used an FDR‐corrected cluster‐wise threshold of *p* < 0.05 in the entire brain space for a voxel‐cluster image generated with a cluster‐defining height threshold of *p* < 0.005, uncorrected. By adopting these approaches, we depicted brain regions in which deactivation was associated with task performance (absolute asynchrony and variance of asynchrony) epoch‐by‐epoch in each participant, which could be consistently observed across all participants in a group (Fig. 4A).

## Results

### Behavioral Results

All of the participants could perform the 1‐Hz movements. The mean numbers of button‐press responses per epoch in the two experimental runs was 14.7 ± 0.2 (standard deviation), 14.8 ± 0.3, and 14.7 ± 0.3 for the CH, ADO, and AD groups, respectively. The one‐way ANOVA showed no significant difference among the groups. Even though the participants were asked to press the button in precise synchronization with the 1‐Hz sounds, the timings of the tones and button‐press were not exactly synchronized. The mean absolute asynchrony was 71.7 ± 35.9 ms, 56.3 ± 20.4 ms, and 57.3 ± 19.6 ms for the CH, ADO, and AD groups, respectively (Fig. 1B). The one‐way ANOVA showed no significant difference among the groups, though the absolute asynchrony tended to be greater in the CH group than in the other groups (Fig. 1B). On the other hand, a significantly greater variance of asynchrony was observed in the CH group. The mean variance of asynchrony was 61.4 ± 14.8 ms, 52.5 ± 16.1 ms, and 48.0 ± 10.1 ms for the CH, ADO, and AD groups, respectively (Fig. 1C). The one‐way ANOVA showed significant group differences [*F* (2, 57) = 4.80, *p* < 0.05], and post‐hoc Bonferroni *t*‐tests revealed significant differences between the CH and AD groups (*p* < 0.01). These results suggest that absolute asynchrony and variance of asynchrony may reflect different aspects of task performance and that children show unstable motor performance in terms of button‐press timing variability.

### Button‐Press‐Related Deactivation in Each Group

We examined button‐press‐related deactivation in every group (Fig. 2). The volume of deactivation appeared to become greater with development (i.e., AD > ADO > CH). In the AD group, we found significant deactivation in broad cortical regions including the ipsilateral (right) SM1 (hand/arm, foot, and face sections), bilateral visual (medial and lateral occipital cortices) and auditory (superior temporal) areas, bilateral lateral occipitoparietal cortices (dorsal visual pathway, including the superior parietal lobules [SPLs] and posterior parietal cortices [PPCs]), medial parietal cortices (cuneus, precuneus, and posterior cingulate cortex [PCC]), ventromedial prefrontal cortices (vMPFCs), and middle temporal gyri (MTGs; Fig. 2 and Supplementary Table 1). The medial parietal cortices, vMPFC, and MTG appeared to correspond well to the brain regions that likely form the DMN (Raichle *et al.*, 2001; Raichle and Snyder, 2007; Mayer *et al.*, 2010; Chai *et al.*, 2014; see more in Discussion). We also found deactivation in the subcortical regions of the right thalamus (e.g., sensorimotor territory) and of the bilateral inferior cerebellar lobules, including the sections previously shown to be functionally connected with the prefrontal cortex, PPC, and DMN (Supplementary Table 1; Habas *et al.*, 2009; O'Reilly *et al.*, 2010; Sang *et al.*, 2012).

In the ADO group, we found similar patterns of deactivation. However, in this group, no apparent deactivation was observed in the bilateral vMPFCs, medial parietal cortices, or medial occipital cortices (Fig. 2). Finally, in the CH group, the volume of deactivation seemed to be smaller than those in the other two groups (Fig. 2). In this group, we only identified significant deactivation in the ipsilateral SM1, bilateral auditory (superior temporal) areas, bilateral SPLs and PPCs, and left inferior cerebellar lobule (Supplementary Table 1).

### Consistent Deactivation Across Groups and Between‐Group Differences

The conjunction analysis revealed that the ipsilateral SM1 (hand/arm and foot sections), bilateral auditory (superior temporal) areas, and bilateral SPLs and PPCs were consistently deactivated across all groups. Thus, these regions are deactivated during the button‐press task since childhood (Fig. 2 and Table 1).

**Table 1:** Results of Conjunction Analysis

Clusters	Size	MNI Coordinates			*T*‐value	Anatomical Identification
		*x*	*y*	*z*		
*Deactivation*						
Right SM1‐SPL‐PPC cluster	3,059	36	−40	64	6.29	Area 1
		36	−34	54	5.58	Area 3b
		24	−54	68	5.31	Area 7PC
		38	−28	48	4.97	Area 4p
		26	−22	56	4.76	Precentral gyrus
		22	−46	70	4.63	Area 5L
		10	−32	50	4.50	Area 5Ci
		28	−60	60	4.43	Area 7A
		18	−74	52	4.14	Area 7P
		32	−6	60	4.00	Superior frontal gyrus
		40	−24	56	3.96	Area 4a (hand)
Left SPL‐PPC cluster	1,136	−24	−42	66	4.39	Area 2
		−8	−62	62	4.23	Area 7A
		−18	−56	66	4.23	Area 5L
		−10	−72	58	3.62	Area 7P
		−10	−66	48	3.57	Precuneus
		−10	−48	56	3.38	Area 5M
		−28	−54	54	3.16	Inferior parietal lobule
		0	−42	66	2.84	Area 4a (foot)
Right superior temporal cluster	665	60	−6	−2	4.83	Superior temporal gyrus
		44	−18	4	4.02	Area TE1.1
		64	−16	2	3.95	Area TE3
		36	−24	16	3.92	Area OP2
		36	−12	18	3.41	Area OP3
		52	−10	8	3.05	Area OP4
		64	−28	−2	3.03	Middle temporal gyrus
Left superior temporal cluster	294	−44	−24	4	4.15	Superior temporal gyrus
		−40	−32	8	3.33	Area TE1.1
		−64	−18	8	3.15	Area TE3
*Activation*						
Right cerebellar cluster	1,110	14	−54	−20	8.07	Lobule VI (Hem)
		2	−50	−4	3.26	Lobule V (Hem)
Left insular cluster	124	−46	0	4	5.01	Insula
Right IFG/insular cluster	222	44	4	2	3.98	Insula
		38	16	10	3.88	IFG (Opercularis)
		54	10	10	3.17	Area 44

For an anatomical identification of peaks, we only considered cytoarchitectonic areas available in the Anatomy toolbox that had >30% probability. The cytoarchitectonic area with the highest probability was reported for each peak. When no cytoarchitectonic area with >30% probability was available to determine a peak, we provided the anatomical location of the peak. In each cluster, we reported peaks that were more than 8 mm apart in the descending *T*‐value order. To facilitate visualization, we avoided reporting a peak for each cluster when it was identified in the cytoarchitectonic area or anatomical structure already reported for a peak with a higher *T*‐value. Uncorrected height threshold, *p < *0.005; extent threshold, *p* < 0.05, false discovery rate (FDR)‐corrected in the entire brain, size = number of active voxels.

Abbreviations: IFG, inferior frontal gyrus; MNI, Montreal Neurological Institute; PPC, posterior parietal cortex; SM1, primary sensorimotor cortex; SPL, superior parietal lobule; Hem, hemisphere.

When we further explored possible developmental changes in brain deactivation, we found that deactivation in a particular brain region progressed in a specific developmental period (Fig. 3 and Table 2). Upon examining possible group differences by one‐way ANOVA, we found significant group differences in the brain regions in the following between‐group comparisons. When compared with that in the CH group, deactivation in the AD group was greater in the ipsilateral SM1 (mainly hand/arm sections), bilateral medial occipital cortices including the primary visual cortices (V1) and auditory association (superior temporal) areas, bilateral medial parietal cortices, vMPFCs, and MTGs (likely forming the DMN), as well as in the right lateral occipitotemporal cortex and bilateral lateral occipitoparietal cortices (Fig. 3A and Table 2).

**Table 2:** Developmental Changes in Brain Deactivation

Clusters	Size	MNI Coordinates			*T*‐value	Anatomical Identification
		*x*	*y*	*z*		
*ADO > CH*						
Right SM1 cluster	188	42	−34	66	4.03	Area 1
		44	−18	64	3.18	Precentral gyrus
Left lateral occipital cluster	188	−16	−90	8	3.38	Superior occipital gyrus
		−44	−86	12	3.38	Area hOc4lp
		−26	−86	−6	2.77	Area hOc4v [V4]
*AD > ADO*						
Bilateral vMPFC cluster	779	−12	58	16	5.23	Superior frontal gyrus
		−2	46	−18	4.08	Area Fp2
		−2	46	8	3.78	ACC
		4	42	−8	3.42	Area s32
		2	60	10	3.33	Area Fp2
		−10	40	−8	3.04	Area s32
Right cuneus cluster	209	20	−62	20	5.02	Cuneus
		12	−64	18	3.48	Calcarine gyrus
Bilateral medial occipito‐parietal cluster	186	0	−80	22	3.66	Area hOc3d [V3d]
		0	−62	24	3.36	Precuneus
		0	−82	32	3.24	Area 7M
*AD > CH*						
Right SM1 cluster (ventral)	219	36	−14	44	4.39	Precentral gyrus
		50	−12	38	3.42	Postcentral gyrus
		44	−16	34	3.30	Area 4P
Right SM1 cluster (dorsal)	154	50	−26	58	3.68	Area 1
		42	−12	64	3.48	Precentral gyrus
		40	−30	62	3.27	Area 4a
Bilateral vMPFC cluster	2,070	−2	44	−20	5.99	Area Fo1
		−14	60	12	5.55	Area Fp1
		−2	60	32	5.38	Superior medial gyrus
		−4	58	−2	5.30	Area Fp2
		−16	40	−14	4.39	Superior orbital gyrus
		2	12	−4	4.36	Area 33
		2	56	−12	4.31	Area Fp2
		−2	44	8	4.26	ACC
		−10	40	−8	4.11	Area s32
Bilateral occipito‐parietal cluster	7,321	−4	−32	40	5.21	MCC
		−8	−44	6	5.10	Calcarine gyrus
		0	−66	22	4.97	Cuneus
		20	−60	18	4.93	Calcarine gyrus
		−10	−48	42	4.83	Precuneus
		6	−78	18	4.79	Area hOc2 [V2]
		2	−78	28	4.28	Area 7M
		−14	−60	0	4.28	Area hOc1 [V1]
		20	−94	28	4.24	Area hOc4d [V3A]
Right lateral occipito‐temporal cluster	163	56	−68	0	3.34	Area hOc4la
Left PPC cluster	325	−46	−70	26	4.11	Area PGp
		−28	−78	48	4.08	Superior parietal lobule
		−20	−80	48	3.68	Area 7P
		−40	−80	40	3.61	Middle occipital gyrus
Left temporal cluster	579	−64	−14	−18	5.14	Middle temporal gyrus
		−62	−20	2	3.35	Area TE3
		−58	−2	−14	3.29	Superior temporal gyrus
		−64	−28	−20	3.02	Inferior temporal gyrus
Right temporal cluster	297	48	−34	−4	4.07	Middle temporal gyrus

See footnote in Table 1. Abbreviations: ACC, anterior cingulate cortex; AD, adults; ADO, adolescents; CH, children; MCC, middle cingulate cortex; MNI, Montreal Neurological Institute; SM1, primary sensorimotor cortex; vMPFC, ventro‐medial prefrontal cortex.

Interestingly, deactivation in the ipsilateral SM1 (hand/arm section) and left lateral occipital cortex (Malikovic *et al.*, 2016) significantly progressed in the ADO group compared to that in the CH group (i.e., from childhood to adolescence; Fig. 3B), while deactivation in the bilateral medial occipital/parietal cortices and vMPFCs progressed in the AD group when compared with that in the ADO group (i.e., from adolescence to adulthood; Fig. 3C).

We did not find any regions with greater deactivation in the CH group compared with those in the other two groups, nor in the ADO group compared with those in the AD group. Thus, regional brain deactivation during the button‐press task progressed with development.

### Relation with Task Performance

When we explored the brain regions in which the degree of deactivation was correlated with the absolute asynchrony, we found that, in the AD group, greater deactivation in the bilateral V1 and medial visual areas was associated with smaller asynchrony (i.e., better performance; Fig. 4 and Table 3). Such correlation was only observed in the AD group. This result suggested that deactivation in the visual areas, progressing from adolescence to adulthood (Fig. 3C), is related to the accuracy of button‐press timing relative to the sound in adults. We did not find any brain regions where deactivation was correlated with larger absolute asynchrony (i.e., worse performance) values in either group. On the other hand, when we explored the brain regions in which the degree of deactivation was correlated with the variance of asynchrony, we did not find any correlation (negative or positive) in either group. This result again supports our view that absolute asynchrony and variance of asynchrony may reflect different aspects of task performance. We speculate that absolute asynchrony may reflect the degree of accuracy in the timing of motor output relative to the sound and could be reduced in a situation where a participant can better focus on performing the task without being distracted by cross‐modal interference from an irrelevant sensory (visual) modality. On the other hand, the variance of asynchrony may reflect trial‐by‐trial variability of motor control, which is suggested to be generated in the neuronal activity of the central motor system (Naito *et al.*, 2016).

**Table 3:** Brain Regions in Which Activity was Correlated with Task Performance in Adults

Clusters	Size	MNI Coordinates			*T*‐value	Anatomical Identification
		*x*	*y*	*z*		
Bilateral medial occipital cluster	535	10	−84	10	4.05	Area hOc1 [V1]
		0	−76	18	3.82	Area hOc2 [V2]
		−14	−66	22	3.61	Precuneus
		−2	−68	18	3.52	Calcarine gyrus
		−2	−98	4	3.15	Area hOc1 [V1]
		−8	−74	18	3.01	Area hOc3d [V3d]

See footnote in Table 1. Abbreviation: MNI, Montreal Neurological Institute.

### Button‐Press‐Related Activation

In the AD group, we found significant button‐press‐related activation (Button‐press > Rest) in the contralateral (left) primary motor cortex (M1; hand/arm section), thalamus (motor territory), and ipsilateral (right) vermal and paravermal sections of the cerebellum (Fig. 2 and Supplementary Table 2). We also found activation in the right inferior frontal cortex, including the anterior insula, and right inferior parietal cortex. The inferior frontoparietal cortices likely form a network connected by the inferior branch of the superior longitudinal fasciculus, and may perform the function of online monitoring of ongoing actions and sensory experiences (Berti *et al.*, 2005; Naito *et al.*, 2017; Morita *et al.*, 2018). In addition to these regions, we also found activation in the left inferior frontal cortex, including the anterior insula, and left cerebellar hemisphere. In the ADO group, a similar pattern of activation was observed (Fig. 2 and Supplementary Table 2). In the CH group, we found a slightly different pattern of brain activation from those in the other two groups (Fig. 2 and Supplementary Table 2).

When we performed a conjunction analysis across all groups, we found consistent brain activation in the ipsilateral (right) vermal and paravermal sections of the cerebellum, right inferior frontal cortex, including the anterior insula, and left anterior insular cortex (Fig. 2 and Table 4). In addition to these regions, we also found small but consistent brain activations in the left SM1, though they did not reach significant levels (Fig. 2). Thus, these brain regions are used in the button‐press task from childhood onward.

**Table 4:** Developmental Changes in Brain Activation

Clusters	Size	MNI Coordinates			*T*‐value	Anatomical Identification
		*x*	*y*	*z*		
*CH > ADO*						
Left SM1 cluster	246	−34	−24	62	5.15	Precentral gyrus
		−32	−26	54	4.98	Area 4p
		−22	−32	56	3.12	Area 3a
*CH > AD*						
Left SM1 cluster	505	−30	−26	52	5.46	Postcentral gyrus
		−34	−24	60	5.37	Precentral gyrus
Bilateral dMPFC cluster	915	−10	60	12	4.63	Superior medial gyrus
		−20	58	14	3.92	Area Fp1
		−8	54	6	3.79	Area Fp2
		−18	34	48	3.76	Superior frontal gyrus
		−12	42	12	3.73	ACC
		8	52	28	3.66	Superior medial gyrus

See footnote in Table 1. Abbreviations: ACC, anterior cingulate cortex; AD, adults; ADO, adolescents; CH, children; dMPFC, dorso‐medial prefrontal cortex; MNI, Montreal Neurological Institute; SM1, primary sensorimotor cortex.

**Figure 5:**
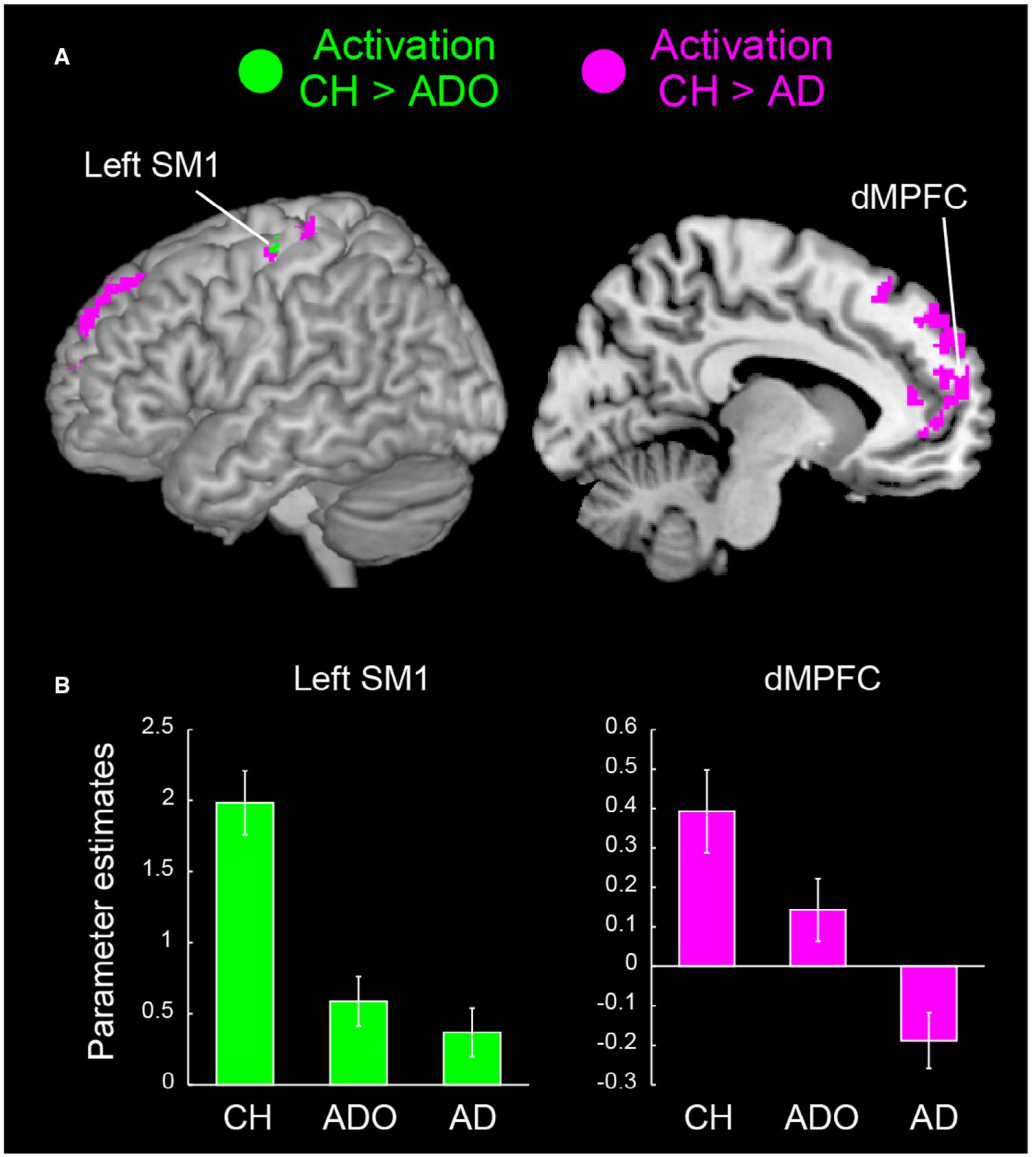
The group differences in button‐press‐related activation and their effect sizes (parameter estimates) in each group. (A) Brain regions in which activation was significantly greater in the CH group than in the AD group (magenta), and in the CH group than in the ADO group (green). Activation was rendered onto the left hemisphere and a sagittal section (*x* = −10) of an MNI standard brain. (B) The mean effect size in each group obtained from the green regions. The mean effect sizes obtained from the contralateral (left) SM1 (peak coordinates = −34, −24, 62) and the dMPFC (−10, 60, 12) are shown. The error bars indicate the standard error of the mean. Abbreviations: AD, adults; ADO, adolescents; CH, children; dMPFC, dorso‐medial prefrontal cortex; MNI, Montreal Neurological Institute; SM1, primary sensorimotor cortex. [Colour figure can be viewed at wileyonlinelibrary.com]

Next, we explored developmental changes in brain activation and found that the contralateral (left) SM1 (hand/arm section) showed significantly greater activation in the CH group than in the ADO and AD groups (Fig. 5A and Table 4). Thus, the sensorimotor activation associated with the execution of a simple button‐press task became weaker and less extensive with development (Figs. 2 and 5B). In the CH vs. AD comparison, we also found that brain activity increased in the bilateral dorsal aspects of the MPFC (dMPFC) in the CH group (Fig. 5A), decreasing with development and eventually turning into deactivation in the AD group (Fig. 5C).

We did not find any brain regions where the degree of activation was correlated with the behavioral indexes (i.e., smaller or greater absolute asynchrony and variance of asynchrony) in any group.

## Discussion

Using a well‐controlled motor task, we explored how brain deactivation is shaped during typical human development and examined whether deactivation may benefit task performance. We demonstrated deactivation in the ipsilateral SM1, cross‐modal (visual and auditory) areas, and DMN in adults with their eyes closed while performing a unimanual button‐press task at a regular pace (Fig. 2). Such deactivation generally progressed with development, albeit deactivation in different brain regions progressed in different developmental periods (Fig. 3). Finally, we showed that cross‐modal deactivation in the bilateral V1 was associated with smaller absolute asynchrony, and therefore better button‐press performance, in adults (Fig. 4). These results suggest that brain deactivation systematically progresses with development and that cross‐modal deactivation develops to benefit some aspects of the task performance in adults.

### Negative BOLD Phenomenon

Physiological mechanisms underlying task‐induced negative BOLD phenomenon (deactivation) are still controversial (Moraschi *et al.*, 2012). However, this phenomenon is thought to be associated with (1) a decrease in CBF caused by (active) neuronal inhibition (Shmuel *et al.*, 2002; 2006; Smith *et al.*, 2004; Pasley *et al.*, 2007; Boorman *et al.*, 2010; Wade and Rowland, 2010; Mullinger *et al*., 2014; Sten *et al*., 2017), (2) a decrease in CBF caused by CBF redistribution (i.e., a blood‐stealing effect, manifesting as hemodynamic changes that have no neural correlates) into nearby active regions (Harel *et al.*, 2002; Devor *et al.*, 2007), (3) an increase in the cerebral metabolic rate of oxygen utilization (CMRO_2_) without a concomitant CBF increase (Schridde *et al.*, 2008), and (4) altered neural regulation of the CBF‐controlling vasculature (Smith *et al.*, 2004; Shih *et al.*, 2009). Importantly, the physiological basis of negative BOLD signaling may depend on the stimulus type (task) and brain region (Kim and Ogawa, 2012).

In the present study, we showed that, in adults performing a regularly paced unimanual motor task, deactivation likely does not occur randomly or uniformly in the entire brain. Instead, we found systematic deactivations in the restricted, task‐irrelevant, and biologically plausible regions (the ipsilateral SM1, visual areas, and DMN). In addition, we demonstrated that deactivation progressed with development, showing region‐specific enhancement in a particular developmental period, despite the participants in all age groups performing the same task. This cannot be merely explained by regional differences in the basal CBF and CMRO_2_ because regional CBF and CMRO_2_ values are higher in children aged 8–16 than in adults, but no significant differences are reported among the frontal, sensorimotor, parietal, temporal, or occipital (visual) areas (Takahashi *et al.*, 1999; Biagi *et al.*, 2007). Finally, we also demonstrated that visual cross‐modal deactivation may benefit motor task performance.

Taken together, our findings indicate that the development of task‐induced deactivation may have physiological significance and is closely linked with the promotion of the segregation of a function required to perform a task, though further neurophysiological investigations are needed to validate this hypothesis.

### Deactivation in the Ipsilateral SM1

We found deactivation in the hand/arm section of the ipsilateral (right) SM1, which was consistently observed across all groups (Fig. 2 and Table 1). Ipsilateral SM1 deactivation has been reported during a unimanual motor task in younger adults (Allison *et al.*, 2000; Newton *et al.*, 2005; Marchand *et al.*, 2007; Hayashi *et al.*, 2008). Similarly, electrical stimulation of the right median nerve may also cause ipsilateral deactivation in the primary somatosensory cortex, which has been proposed to reflect functional inhibition in the somatosensory system (Kastrup *et al.*, 2008; Klingner *et al.*, 2010; 2015; Schäfer *et al.*, 2012; Gröschel *et al.*, 2013).

M1 activity can be suppressed by interhemispheric (transcallosal) inhibition exerted from the opposite side M1 (Ferbert *et al.*, 1992) to reduce its superfluous activity (Kobayashi *et al.*, 2003). In older adults, it is shown that reduced interhemispheric inhibition exerted from the contralateral (left) M1 (Talelli *et al.*, 2008) may contribute to the aging‐related reduction in ipsilateral (right) M1 deactivation during unimanual right hand motor tasks (Hutchinson *et al.*, 2002; Naccarato *et al.*, 2006; Riecker *et al.*, 2006; Ward *et al.*, 2008; Loibl *et al.*, 2011). These results, together with the neurophysiological evidence from an animal study (Palmer *et al.*, 2012), allow us to assume that interhemispheric inhibition exerted from the left (contralateral) SM1 contributed, at least partially, to the deactivation of the ipsilateral SM1 hand section in the present study, suppressing superfluous ipsilateral activation during right hand finger movements (cf. Geffen *et al.*, 1994), though we cannot exclude the possibility of inhibition from other brain structures (cf. Blankenburg *et al.*, 2003).

Even though ipsilateral SM1 deactivation was already observable during childhood (Fig. 2), it became more prominent during adolescence (Fig. 3 and Table 2). Indeed, ipsilateral SM1 deactivation gradually increased from childhood to adolescence (Supplementary Figure 1B). This developmental period appears to correspond well with the suppression of executive function timing implemented in the ipsilateral M1 by the development of inhibitory systems, such as transcallosal inhibition from the contralateral M1 (Hanakawa *et al.*, 2005). If the ipsilateral SM1 deactivation is associated with the development of interhemispheric inhibition during the childhood‐to‐adolescence transition, this result is compatible with our previous result showing the emergence of right‐hemispheric dominance in bodily recognition during adolescence by suppressing the activity in the left hemisphere (Naito *et al.*, 2017). The transition to adolescence appears to be a developmental period when lateralized use of cerebral hemispheres is facilitated for some brain functions (at least for bodily recognition and unimanual movements).

Another interesting finding is that the ipsilateral paracentral deactivation is consistently observed across all age groups (see medial view in Fig. 2 and Table 1). Such deactivation has only been described occasionally during a unimanual right hand finger motor task in adults (Kudo *et al.*, 2004). The involved brain region likely corresponds to the foot section of the SM1 (Ehrsson *et al.*, 2003; Naito *et al.*, 2007; Naito and Hirose, 2014). Together with deactivation in the putative face section of the ipsilateral SM1 in the ADO and AD groups (Fig. 2; Ehrsson *et al.*, 2003; Hanakawa *et al.*, 2005), this result implies the possibility of cross‐somatotopical (from the hand section to the foot and face sections) inhibition within the SM1, which may help to functionally suppress the movements of irrelevant body parts as also suggested in animal studies (Jacobs and Donoghue, 1991; Schneider *et al.*, 2002).

### Deactivation in Visual and Auditory Areas (Cross‐Modal Inhibition)

We found deactivation in the visual and auditory areas during the hand motor task performed with no vision. Such deactivation can be considered cross‐modal inhibition, previously observed in the adult brain in numerous studies (see the references in the Introduction).

The suppression of brain activity in the visual areas may prevent cross‐modal distraction and interference from the irrelevant sensory modality, allowing the brain to focus on precise motor performance (cf. Ghatan *et al.*, 1998). Although deactivation in the visual (occipital) cortex have been repeatedly shown in blindfolded adults performing somatosensory (tactile) discrimination tasks (Kawashima *et al.*, 1995; Sadato *et al.*, 1996; 1998; Weisser *et al.*, 2005; Merabet *et al.*, 2007), such deactivation has only been described occasionally during hand motor tasks (Jäncke *et al.*, 2000; Hou *et al.*, 2016). Thus, the present study confirmed the existence of substantial deactivation in the visual cortex and showed, for the first time, its development during a motor task.

The visual deactivation we observed covered both the lateral and medial aspects of the occipital cortex and extended dorsally into the PPCs and SPLs of both hemispheres (Fig. 2). Hence, the deactivation was most likely located in the dorsal visual pathway (Goodale and Milner, 1992). One of our interesting findings is that the parietal (SPL and PPC) regions are already deactivated during childhood and become further deactivated with increasing age (Supplementary Figure 1). The human SPL and PPC are generally considered as visuospatial parietal association cortices, which are involved in a wide range of visuomotor tasks such as reaching (Connolly *et al.*, 2003; Culham and Valyear, 2006; Filimon, 2010). These regions are connected with the SM1 in the primate brain (Darian‐Smith *et al.*, 1993; Stepniewska *et al.*, 1993; Scheperjans *et al.*, 2005). Hence, it appears that, starting in childhood, the brain is capable of suppressing neuronal activity in these motor‐related parietal regions during a motor task that does not strictly require visual information.

Another important finding is that deactivation in the dorsal visual pathway appeared to expand from the parietal cortices (during childhood) to the lateral occipital cortices (during adolescence) and eventually to the medial occipital cortices including V1 (during adulthood; Figs. 2 and 3 and Tables 1 and 2). Hence, deactivation in the dorsal visual pathway appears to develop and expand from the hierarchically higher parietal association areas to the lower occipital visual regions.

An immediate question arises of how the motor system exerts cross‐modal inhibition in remote regions of the dorsal visual pathway; answering this query requires further study. However, recent animal studies have shown the existence of cross‐modal inhibition both in visual (Leinweber *et al.*, 2017) and auditory (Schneider *et al.*, 2018) cortices from motor areas, and evidence exists in humans that transcranial magnetic stimulation of the human frontal cortex (e.g., the frontal eye field) may generate a negative BOLD signal in remote brain regions such as the visual cortex (Ruff *et al.*, 2006; 2008; 2009). These results may imply the possibility of the existence of long‐distance, cross‐cortex pathways (see also Blankenburg *et al.*, 2008) that can generate inhibitory effects in the projected brain regions.

Perhaps most importantly, deactivation in the bilateral V1 was associated with better motor performance (smaller absolute asynchrony; Fig. 4 and Table 3). Critically, this deactivation was only observed in adults. The cross‐modal deactivation in the V1 progressed prominently in the transition from adolescence to adulthood (Figs. 2 and 3); this deactivation benefits motor task performance during adulthood (Fig. 4). We assume that visual deactivation allows the mature brain to better focus on performing a motor task when one's eyes are closed, by avoiding cross‐modal distraction of the sensorimotor system by an irrelevant sensory (visual) modality.

We also found deactivation in the bilateral superior temporal gyri, including cytoarchitectonic areas TE 1.1 and 3, consistently across all groups (Fig. 2). These regions correspond to the auditory and auditory association cortices (Morosan *et al.*, 2001). Motor‐induced suppression of the auditory cortex has been reported during simple finger tapping tasks (Jäncke *et al.*, 2000; Aliu *et al.*, 2009), consistent with our finding of cross‐modal inhibition. Similar to visual deactivation, auditory deactivation also increased with development (Fig. 3). However, unlike visual cortices, auditory deactivation was already observable during childhood (Fig. 2).

The present task was an auditory paced button‐press task; hence, auditory processing was expected to be relevant to performing the task. However, we found deactivation in the auditory cortex. In addition, we confirmed in our control experiment on another group of adult participants that different pitches in the sounds between the button‐press epochs and the resting periods under the noisy scanning environment did not create any significant auditory deactivations during the button‐press epochs. We used a 1‐Hz sound as a pacemaker for button‐press. The 1‐Hz regularity likely enables the participants to predict the timing of its component tones. Indeed, when we evaluated absolute asynchrony, it was substantially smaller than the reaction time (RT) normally observed in a simple auditory RT task (Fig. 1B; Naito *et al.*, 2000), suggesting that the participants could generate the 1‐Hz movements predictively, largely without relying on the auditory cue. Thus, the task did not depend on demanding auditory processing, and therefore is likely to reveal cross‐modal deactivation of the auditory by the motor system.

### Deactivation in the DMN

We found robust deactivation in the medial parietal cortices, vMPFC, and MTG in adults (Figs. 2 and 3), and these areas appeared to closely overlap the main constituents of the DMN (Raichle *et al.*, 2001; Raichle and Snyder, 2007; Mayer *et al.*, 2010; Chai *et al.*, 2014). It is widely accepted that these brain regions are usually active in a resting condition and deactivated during a variety of goal‐oriented tasks (Mazoyer *et al.*, 2001; Raichle *et al.*, 2001; Binder, 2012). The DMN deactivation observed during the motor task in adults is in agreement with the previous reports of DMN deactivation during unimanual finger motor tasks (Kudo *et al.*, 2004; Marchand *et al.*, 2007) and during manual tactile discrimination tasks (Sadato *et al.*, 1996; Weisser *et al.*, 2005). Importantly, unlike ipsilateral SM1 deactivation, medial parietal, and vMPFC deactivation emerged prominently during adulthood (Figs. 2 and 3 and Table 2), suggesting that motor‐induced deactivation develops in the DMN slowly.

It is shown that the default regions are only sparsely connected functionally at an early school age (7–9 years), and these regions integrate into a cohesive, interconnected network during further development (Fair *et al.*, 2008), though an adult‐like DMN pattern was reported to be already present in the resting‐state brains of children aged 2 (Gao *et al.*, 2015) and 5–8 (de Bie *et al.*, 2012). We found no prominent motor‐induced DMN deactivation during childhood, which is compatible with no prominent DMN deactivation during memory encoding in children (Chai *et al.*, 2014). On the other hand, task‐induced deactivation in the DMN (medial parietal and vMPFC) is reported in 7–12‐year‐old children during a working memory (WM) task (Thomason *et al.*, 2008). Hence, DMN deactivation in children appears to be task‐dependent.

As for the relationship between DMN deactivation and task performance, greater DMN deactivation during a WM task in adults was reported to be associated with the correct encoding in the task (Daselaar *et al.*, 2004; Anticevic *et al.*, 2010; 2012). In addition, pharmacological augmentation of vMPFC deactivation may facilitate the performance of simple visuomotor RT tasks (Minzenberg *et al.*, 2011). In our motor task analysis, we found no voxels in the DMN showing a correlation between the degree of deactivation and task performance. These results indicate that motor‐induced DMN deactivation may reflect motor‐specific neuronal processes distinct from those involved in WM tasks (see also Marchand *et al.*, 2007).

### Development of Brain Activation

The activation in the hand section of the contralateral SM1 became weaker and less extensive with increasing age (Figs. 2 and 5B, and Supplementary Figure 1B). The greater contralateral SM1 activation in children than in adults (Fig. 5) is in line with previous reports from other unimanual finger tapping tasks (De Guio *et al.*, 2012; Turesky *et al.*, 2018). Exact neuronal underpinning of this phenomenon is still unknown; however, the extensive activation in children might reflect their broader index finger representation in the SM1 as their somatotopical representations are likely under development (Nebel *et al.*, 2014). The age‐dependent attenuation of SM1 activation also resembles use‐dependent plasticity in the SM1, which can be observed in professional pianists (Krings *et al.*, 2000) and sport athletes, such as the Brazilian footballer Neymar (Naito and Hirose, 2014). A non‐human primate study revealed that years of motor training may increase the synaptic efficiency in the M1 such that cortical motor neurons can still fire effectively when receiving smaller (limited) amounts of synaptic input during the well‐trained motor task (Picard *et al.*, 2013). Since a BOLD signal reflects synaptic activity (Logothetis *et al.*, 2001), the present age‐dependent attenuation of SM1 activation could be associated with an age‐dependent increase in synaptic efficiency in the contralateral (left) M1 hand section through the long‐term use of right hand fingers during routine daily manual tasks. This view is compatible with the idea that greater neuronal resources (e.g., synaptic inputs) are generally required in children performing the same task as adults (De Guio *et al.*, 2012; Turesky *et al.*, 2018). If our view is correct, the smaller and weaker BOLD signal in the SM1 during a motor task is not specific to pianists’ and athletes’ brains, but is also observable in typical human development.

Another interesting finding of our study is the task‐induced increased activity of the dMPFC during childhood, which is eventually deactivated during adulthood (Fig. 5B). Similar MPFC activation was reported in children experiencing an illusory foot movement (Fontan *et al.*, 2017). Our task and the motor illusion task both require monitoring of ongoing actions and proprioceptive experiences. Hence, our result suggests that the child's brain may activate the MPFC during a task that requires monitoring of sensory‐motor events in his/her body space. The MPFC is generally known to be involved in self‐referential (reflective) processing, such as introspection of one's own thoughts, personal traits, and emotional states (Decety and Sommerville, 2003; Vogeley and Fink, 2003; Macrae *et al.*, 2004; Ochsner *et al.*, 2005; Amodio and Frith, 2006; Northoff *et al.*, 2006). Importantly, children and adolescents have been shown to activate the MPFC during introspective self‐related processing more strongly than adults (Pfeifer *et al.*, 2007; Sebastian *et al.*, 2008). A child's brain thus appears to utilize the MPFC not only for metaphysical but also for physical self‐related processing, indicating immaturity of the functional segregation in the MPFC.

### Conclusion

Using a motor task, we demonstrated that task‐induced deactivation generally progressed with development, and deactivation in certain brain regions progressed during a particular developmental period. The results suggest that task‐induced brain deactivation systematically progresses with development. The present study may elucidate functional differentiation processes of a human brain (motor) system in terms of brain deactivation that have never been systematically described.

## Data Availability

The data that support the findings of this study are available from the corresponding author upon reasonable request.

## References

[dneu22701-bib-0001] Aliu, S.O. , Houde, J.F. and Nagarajan, S.S. (2009) Motor induced suppression of auditory cortex. Journal of Cognitive Neuroscience, 21, 791–802. 18593265 10.1162/jocn.2009.21055 PMC2944400

[dneu22701-bib-0002] Allison, J.D. , Meador, K.J. , Loring, D.W. , Figueroa, R.E. and Wright, J.C. (2000) Functional MRI cerebral activation and deactivation during finger movement. Neurology, 54, 135–142. 10636139 10.1212/wnl.54.1.135

[dneu22701-bib-0003] Amedi, A. , Malach, R. and Pascual‐Leone, A. (2005) Negative bold differentiates visual imagery and perception. Neuron, 48, 859–872. 16337922 10.1016/j.neuron.2005.10.032

[dneu22701-bib-0004] Amodio, D.M. and Frith, C.D. (2006) Meeting of minds: the medial frontal cortex and social cognition. Nature Reviews Neuroscience, 7, 268–277. 16552413 10.1038/nrn1884

[dneu22701-bib-0005] Anticevic, A. , Cole, M.W. , Murray, J.D. , Corlett, P.R. , Wang, X.J. and Krystal, J.H. (2012) The role of default network deactivation in cognition and disease. Trends in Cognitive Sciences, 16, 584–592. 23142417 10.1016/j.tics.2012.10.008 PMC3501603

[dneu22701-bib-0006] Anticevic, A. , Repovs, G. , Shulman, G.L. and Barch, D.M. (2010) When less is more: TPJ and default network deactivation during encoding predicts working memory performance. NeuroImage, 49, 2638–2648. 19913622 10.1016/j.neuroimage.2009.11.008 PMC3226712

[dneu22701-bib-0007] Berti, A. , Bottini, G. , Gandola, M. , Pia, L. , Smania, N. , Stracciari, A. *et al.* (2005) Shared cortical anatomy for motor awareness and motor control. Science, 309, 488–491. 16020740 10.1126/science.1110625

[dneu22701-bib-0008] Biagi, L. , Abbruzzese, A. , Bianchi, M.C. , Alsop, D.C. , Del Guerra, A. and Tosetti, M. (2007) Age dependence of cerebral perfusion assessed by magnetic resonance continuous arterial spin labeling. Journal of Magnetic Resonance Imaging: An Official Journal of the International Society for Magnetic Resonance in Medicine, 25, 696–702. 10.1002/jmri.20839 17279531

[dneu22701-bib-0009] de Bie, H.M. , Boersma, M. , Adriaanse, S. , Veltman, D.J. , Wink, A.M. , Roosendaal, S.D. *et al.* (2012) Resting‐state networks in awake five‐ to eight‐year old children. Human Brain Mapping, 33, 1189–1201. 21520347 10.1002/hbm.21280 PMC6870031

[dneu22701-bib-0010] Binder, J.R. (2012) Task‐induced deactivation and the "resting" state. NeuroImage, 62, 1086–1091. 21979380 10.1016/j.neuroimage.2011.09.026 PMC3389183

[dneu22701-bib-0011] Blankenburg, F. , Ruff, C.C. , Bestmann, S. , Bjoertomt, O. , Eshel, N. , Josephs, O. *et al.* (2008) Interhemispheric effect of parietal TMS on somatosensory response confirmed directly with concurrent TMS‐fMRI. Journal of Neuroscience, 28, 13202–13208. 19052211 10.1523/JNEUROSCI.3043-08.2008 PMC2600426

[dneu22701-bib-0012] Blankenburg, F. , Taskin, B. , Ruben, J. , Moosmann, M. , Ritter, P. , Curio, G. *et al.* (2003) Imperceptible stimuli and sensory processing impediment. Science, 299, 1864. 12649475 10.1126/science.1080806

[dneu22701-bib-0013] Boorman, L. , Kennerley, A.J. , Johnston, D. , Jones, M. , Zheng, Y. , Redgrave, P. *et al.* (2010) Negative blood oxygen level dependence in the rat: a model for investigating the role of suppression in neurovascular coupling. Journal of Neuroscience, 30, 4285–4294. 20335464 10.1523/JNEUROSCI.6063-09.2010 PMC6634501

[dneu22701-bib-0014] Büchel, C. , Holmes, A.P. , Rees, G. and Friston, K.J. (1998) Characterizing stimulus‐response functions using nonlinear regressors in parametric fMRI experiments. NeuroImage, 8, 140–148. 9740757 10.1006/nimg.1998.0351

[dneu22701-bib-0015] Chai, X.J. , Ofen, N. , Gabrieli, J.D. and Whitfield‐Gabrieli, S. (2014) Development of deactivation of the default‐mode network during episodic memory formation. NeuroImage, 84, 932–938. 24064072 10.1016/j.neuroimage.2013.09.032 PMC4175451

[dneu22701-bib-0016] Connolly, J.D. , Andersen, R.A. and Goodale, M.A. (2003) FMRI evidence for a ‘parietal reach region’ in the human brain. Experimental Brain Research, 153, 140–145. 12955383 10.1007/s00221-003-1587-1

[dneu22701-bib-0017] Culham, J.C. and Valyear, H.F. (2006) Human parietal cortex in action. Current Opinion in Neurobiology, 16, 205–212. 16563735 10.1016/j.conb.2006.03.005

[dneu22701-bib-0018] Darian‐Smith, C. , Darian‐Smith, I. , Burman, K. and Ratcliffe, N. (1993) Ipsilateral cortical projections to areas 3a, 3b, and 4 in the macaque monkey. Journal of Comparative Neurology, 335, 200–213. 8227514 10.1002/cne.903350205

[dneu22701-bib-0019] Daselaar, S.M. , Prince, S.E. and Cabeza, R. (2004) When less means more: deactivations during encoding that predict subsequent memory. NeuroImage, 23, 921–927. 15528092 10.1016/j.neuroimage.2004.07.031

[dneu22701-bib-0020] De Guio, F. , Jacobson, S.W. , Molteno, C.D. , Jacobson, J.L. and Meintjes, E.M. (2012) An fMRI study comparing rhythmic finger tapping in children and adults. Pediatric Neurology, 46, 94–100. 22264703 10.1016/j.pediatrneurol.2011.11.019 PMC3266619

[dneu22701-bib-0021] Decety, J. and Sommerville, J.A. (2003) Shared representations between self and other: a social cognitive neuroscience view. Trends in Cognitive Sciences, 7, 527–533. 14643368 10.1016/j.tics.2003.10.004

[dneu22701-bib-0022] Devor, A. , Tian, P. , Nishimura, N. , Teng, I.C. , Hillman, E.M.C. , Narayanan, S.N. *et al.* (2007) Suppressed neuronal activity and concurrent arteriolar vasoconstriction may explain negative blood oxygenation level‐dependent signal. Journal of Neuroscience, 27, 4452–4459. 17442830 10.1523/JNEUROSCI.0134-07.2007 PMC2680207

[dneu22701-bib-0023] Ehrsson, H.H. , Geyer, S. and Naito, E. (2003) Imagery of voluntary movement of fingers, toes, and tongue activates corresponding body‐part‐specific motor representations. Journal of Neurophysiology, 90, 3304–3316. 14615433 10.1152/jn.01113.2002

[dneu22701-bib-0024] Eickhoff, S.B. , Stephan, K.E. , Mohlberg, H. , Grefkes, C. , Fink, G.R. , Amunts, K. *et al.* (2005) A new SPM toolbox for combining probabilistic cytoarchitectonic maps and functional imaging data. NeuroImage, 25, 1325–1335. 15850749 10.1016/j.neuroimage.2004.12.034

[dneu22701-bib-0025] Engelhardt, L.E. , Roe, M.A. , Juranek, J. , DeMaster, D. , Harden, K.P. , Tucker‐Drob, E.M. *et al.* (2017) Children's head motion during fMRI tasks is heritable and stable over time. Developmental Cognitive Neuroscience, 25, 58–68. 28223034 10.1016/j.dcn.2017.01.011 PMC5478437

[dneu22701-bib-0026] Evans, A.C. , Kamber, M. , Collins, D.L. and MacDonald, D. (1994) An MRI‐based probabilistic atlas of neuroanatomy In: Shorvon S.D., Fish D.R., Andermann F., Bydder G.M. and Stefan H. (Eds.) Magnetic Resonance Scanning and Epilepsy. Boston, MA: Springer, Vol. 264, pp. 263–274.

[dneu22701-bib-0027] Fair, D.A. , Cohen, A.L. , Dosenbach, N.U. , Church, J.A. , Miezin, F.M. , Barch, D.M. *et al.* (2008) The maturing architecture of the brain's default network. Proceedings of the National Academy of Sciences, 105, 4028–4032. 10.1073/pnas.0800376105 PMC2268790 18322013

[dneu22701-bib-0028] Ferbert, A. , Priori, A. , Rothwell, J.C. , Day, B.L. , Colebatch, J.G. and Marsden, C.D. (1992) Interhemispheric inhibition of the human motor cortex. The Journal of Physiology, 453, 525–546. 1464843 10.1113/jphysiol.1992.sp019243 PMC1175572

[dneu22701-bib-0029] Filimon, F. (2010) Human cortical control of hand movements: parietofrontal networks for reaching, grasping, and pointing. The Neuroscientist, 16, 388–407. 20817917 10.1177/1073858410375468

[dneu22701-bib-0030] Fontan, A. , Cignetti, F. , Nazarian, B. , Anton, J.L. , Vaugoyeau, M. and Assaiante, C. (2017) How does the body representation system develop in the human brain? Developmental Cognitive Neuroscience, 24, 118–128. 28314184 10.1016/j.dcn.2017.02.010 PMC6987789

[dneu22701-bib-0031] Friston, K.J. , Holmes, A.P. , Poline, J.B. , Grasby, P.J. , Williams, S.C. , Frackowiak, R.S. *et al.* (1995) Analysis of fMRI time‐series revisited. NeuroImage, 2, 45–53. 9343589 10.1006/nimg.1995.1007

[dneu22701-bib-0032] Gao, W. , Alcauter, S. , Smith, J.K. , Gilmore, J.H. and Lin, W. (2015) Development of human brain cortical network architecture during infancy. Brain Structure and Function, 220, 1173–1186. 24469153 10.1007/s00429-014-0710-3 PMC4850360

[dneu22701-bib-0033] Geffen, G.M. , Jones, D.L. and Geffen, L.B. (1994) Interhemispheric control of manual motor activity. Behavioural Brain Research, 64, 131–140. 7840879 10.1016/0166-4328(94)90125-2

[dneu22701-bib-0034] Genovese, C.R. , Lazar, N.A. and Nichols, T. (2002) Thresholding of statistical maps in functional neuroimaging using the false discovery rate. NeuroImage, 15, 870–878. 11906227 10.1006/nimg.2001.1037

[dneu22701-bib-0035] Ghatan, P.H. , Hsieh, J.C. , Petersson, K.M. , Stone‐Elander, S. and Ingvar, M. (1998) Coexistence of attention‐based facilitation and inhibition in the human cortex. NeuroImage, 7, 23–29. 9500831 10.1006/nimg.1997.0307

[dneu22701-bib-0036] Goodale, M.A. and Milner, A.D. (1992) Separate visual pathways for perception and action. Trends in Neurosciences, 15, 20–25. 1374953 10.1016/0166-2236(92)90344-8

[dneu22701-bib-0037] Gröschel, S. , Sohns, J.M. , Schmidt‐Samoa, C. , Baudewig, J. , Becker, L. , Dechent, P. *et al.* (2013) Effects of age on negative BOLD signal changes in the primary somatosensory cortex. NeuroImage, 71, 10–18. 23296182 10.1016/j.neuroimage.2012.12.039

[dneu22701-bib-0038] Habas, C. , Kamdar, N. , Nguyen, D. , Prater, K. , Beckmann, C.F. , Menon, V. *et al.* (2009) Distinct cerebellar contributions to intrinsic connectivity networks. Journal of Neuroscience, 29, 8586–8594. 19571149 10.1523/JNEUROSCI.1868-09.2009 PMC2742620

[dneu22701-bib-0039] Hanakawa, T. , Parikh, S. , Bruno, M.K. and Hallett, M. (2005) Finger and face representations in the ipsilateral precentral motor areas in humans. Journal of Neurophysiology, 93, 2950–2958. 15625099 10.1152/jn.00784.2004 PMC1440886

[dneu22701-bib-0040] Harel, N. , Lee, S.P. , Nagaoka, T. , Kim, D.S. and Kim, S.G. (2002) Origin of negative blood oxygenation level‐dependent fMRI signals. Journal of Cerebral Blood Flow & Metabolism, 22, 908–917. 12172376 10.1097/00004647-200208000-00002

[dneu22701-bib-0041] Haxby, J.V. , Horwitz, B. , Ungerleider, L.G. , Maisog, J.M. , Pietrini, P. and Grady, C.L. (1994) The functional organization of human extrastriate cortex: a PET‐rCBF study of selective attention to faces and locations. Journal of Neuroscience, 14, 6336–6353. 7965040 10.1523/JNEUROSCI.14-11-06336.1994 PMC6577268

[dneu22701-bib-0042] Hayashi, M.J. , Saito, D.N. , Aramaki, Y. , Asai, T. , Fujibayashi, Y. and Sadato, N. (2008) Hemispheric asymmetry of frequency‐dependent suppression in the ipsilateral primary motor cortex during finger movement: a functional magnetic resonance imaging study. Cerebral Cortex, 18, 2932–2940. 18413350 10.1093/cercor/bhn053 PMC2583153

[dneu22701-bib-0043] Holmes, A.P. and Friston, K.J. (1998) Generalisability, random effects and population inference. NeuroImage, 7, S754.

[dneu22701-bib-0044] Hou, L.J. , Song, Z. , Pan, Z.J. , Cheng, J.L. , Yu, Y. and Wang, J. (2016) Decreased activation of subcortical brain areas in the motor fatigue state: an fMRI study. Frontiers in Psychology, 7, 1154. 27536264 10.3389/fpsyg.2016.01154 PMC4971080

[dneu22701-bib-0045] Hutchinson, S. , Kobayashi, M. , Horkan, C.M. , Pascual‐Leone, A. , Alexander, M.P. and Schlaug, G. (2002) Age‐related differences in movement representation. NeuroImage, 17, 1720–1728. 12498746 10.1006/nimg.2002.1309

[dneu22701-bib-0046] Jacobs, K.M. and Donoghue, J.P. (1991) Reshaping the cortical motor map by unmasking latent intracortical connections. Science, 251, 944–947. 2000496 10.1126/science.2000496

[dneu22701-bib-0047] Jäncke, L. , Loose, R. , Lutz, K. , Specht, K. and Shah, N.J. (2000) Cortical activations during paced finger‐tapping applying visual and auditory pacing stimuli. Cognitive Brain Research, 10, 51–66. 10978692 10.1016/s0926-6410(00)00022-7

[dneu22701-bib-0048] Jorge, J. , Figueiredo, P. , Gruetter, R. and van der Zwaag, W. (2018) Mapping and characterization of positive and negative BOLD responses to visual stimulation in multiple brain regions at 7T. Human Brain Mapping, 39, 2426–2441. 29464809 10.1002/hbm.24012 PMC6866646

[dneu22701-bib-0049] Kang, H.C. , Burgund, E.D. , Lugar, H.M. , Petersen, S.E. and Schlaggara, B.L. (2003) Comparison of functional activation foci in children and adults using a common stereotactic space. NeuroImage, 19, 16–28. 12781724 10.1016/s1053-8119(03)00038-7

[dneu22701-bib-0050] Kastrup, A. , Baudewig, J. , Schnaudigel, S. , Huonker, R. , Becker, L. , Sohns, J.M. *et al.* (2008) Behavioral correlates of negative BOLD signal changes in the primary somatosensory cortex. NeuroImage, 41, 1364–1371. 18495495 10.1016/j.neuroimage.2008.03.049

[dneu22701-bib-0051] Kawashima, R. , O'Sullivan, B.T. and Roland, P.E. (1995) Positron‐emission tomography studies of cross‐modality inhibition in selective attentional tasks: closing the “mind's eye”. Proceedings of the National Academy of Sciences, 92, 5969–5972. 10.1073/pnas.92.13.5969 PMC41623 7597062

[dneu22701-bib-0052] Kim, S.G. and Ogawa, S. (2012) Biophysical and physiological origins of blood oxygenation level‐dependent fMRI signals. Journal of Cerebral Blood Flow & Metabolism, 32, 1188–1206. 22395207 10.1038/jcbfm.2012.23 PMC3390806

[dneu22701-bib-0053] Klingner, C.M. , Brodoehl, S. and Witte, O.W. (2015) The importance of the negative blood‐oxygenation‐level‐dependent (BOLD) response in the somatosensory cortex. Reviews in the Neurosciences, 26, 647–653. 26057216 10.1515/revneuro-2015-0002

[dneu22701-bib-0054] Klingner, C.M. , Hasler, C. , Brodoehl, S. and Witte, O.W. (2010) Dependence of the negative BOLD response on somatosensory stimulus intensity. NeuroImage, 53, 189–195. 20538064 10.1016/j.neuroimage.2010.05.087

[dneu22701-bib-0055] Kobayashi, M. , Hutchinson, S. , Schlaug, G. and Pascual‐Leone, A. (2003) Ipsilateral motor cortex activation on functional magnetic resonance imaging during unilateral hand movements is related to interhemispheric interactions. NeuroImage, 20, 2259–2270. 14683727 10.1016/s1053-8119(03)00220-9

[dneu22701-bib-0056] Kriegeskorte, N. , Simmons, W.K. , Bellgowan, P.S. and Baker, C.I. (2009) Circular analysis in systems neuroscience: the dangers of double dipping. Nature Neuroscience, 12, 535–540. 19396166 10.1038/nn.2303 PMC2841687

[dneu22701-bib-0057] Krings, T. , Töpper, R. , Foltys, H. , Erberich, S. , Sparing, R. , Willmes, K. *et al.* (2000) Cortical activation patterns during complex motor tasks in piano players and control subjects. A functional magnetic resonance imaging study. Neuroscience Letters, 278, 189–193. 10653025 10.1016/s0304-3940(99)00930-1

[dneu22701-bib-0058] Kudo, K. , Miyazaki, M. , Kimura, T. , Yamanaka, K. , Kadota, H. , Hirashima, M. *et al.* (2004) Selective activation and deactivation of the human brain structures between speeded and precisely timed tapping responses to identical visual stimulus: an fMRI study. NeuroImage, 22, 1291–1301. 15219601 10.1016/j.neuroimage.2004.03.043

[dneu22701-bib-0059] Laurienti, P.J. , Burdette, J.H. , Wallace, M.T. , Yen, Y.F. , Field, A.S. and Stein, B.E. (2002) Deactivation of sensory‐specific cortex by cross‐modal stimuli. Journal of Cognitive Neuroscience, 14, 420–429. 11970801 10.1162/089892902317361930

[dneu22701-bib-0060] Leinweber, M. , Ward, D.R. , Sobczak, J.M. , Attinger, A. and Keller, G.B. (2017) A sensorimotor circuit in mouse cortex for visual flow predictions. Neuron, 96, 1204. 10.1016/j.neuron.2017.11.009 29216453

[dneu22701-bib-0061] Lewis, J.W. , Beauchamp, M.S. and DeYoe, E.A. (2000) A comparison of visual and auditory motion processing in human cerebral cortex. Cerebral Cortex, 10, 873–888. 10982748 10.1093/cercor/10.9.873

[dneu22701-bib-0062] Lewis, P.A. , Wing, A.M. , Pope, P.A. , Praamstra, P. and Miall, R.C. (2004) Brain activity correlates differentially with increasing temporal complexity of rhythms during initialization, synchronization, and continuation phases of paced finger tapping. Neuropsychologia, 42, 1301–1312. 15193939 10.1016/j.neuropsychologia.2004.03.001

[dneu22701-bib-0063] Logothetis, N.K. , Pauls, J. , Augath, M. , Trinath, T. and Oeltermann, A. (2001) Neurophysiological investigation of the basis of the fMRI signal. Nature, 412, 150–157. 11449264 10.1038/35084005

[dneu22701-bib-0064] Loibl, M. , Beutling, W. , Kaza, E. and Lotze, M. (2011) Non‐effective increase of fMRI‐activation for motor performance in elder individuals. Behavioural Brain Research, 223, 280–286. 21569800 10.1016/j.bbr.2011.04.040

[dneu22701-bib-0065] Macrae, C.N. , Moran, J.M. , Heatherton, T.F. , Banfield, J.F. and Kelley, W.M. (2004) Medial prefrontal activity predicts memory of self. Cerebral Cortex, 14, 647–654. 15084488 10.1093/cercor/bhh025

[dneu22701-bib-0066] Malikovic, A. , Amunts, K. , Schleicher, A. , Mohlberg, H. , Kujovic, M. , Palomero‐Gallagher, N. *et al.* (2016) Cytoarchitecture of the human lateral occipital cortex: mapping of two extrastriate areas hOc4la and hOc4lp. Brain Structure and Function, 221, 1877–1897. 25687261 10.1007/s00429-015-1009-8

[dneu22701-bib-0067] Marchand, W.R. , Lee, J.N. , Thatcher, J.W. , Thatcher, G.W. , Jensen, C. and Starr, J. (2007) Motor deactivation in the human cortex and basal ganglia. NeuroImage, 38, 538–548. 17888686 10.1016/j.neuroimage.2007.07.036

[dneu22701-bib-0068] Mayer, J.S. , Roebroeck, A. , Maurer, K. and Linden, D.E. (2010) Specialization in the default mode: task‐induced brain deactivations dissociate between visual working memory and attention. Human Brain Mapping, 31, 126–139. 19639552 10.1002/hbm.20850 PMC6870780

[dneu22701-bib-0069] Mazoyer, B. , Zago, L. , Mellet, E. , Bricogne, S. , Etard, O. , Houdé, O. *et al.* (2001) Cortical networks for working memory and executive functions sustain the conscious resting state in man. Brain Research Bulletin, 54, 287–298. 11287133 10.1016/s0361-9230(00)00437-8

[dneu22701-bib-0070] McKiernan, K.A. , D'angelo, B.R. , Kaufman, J.N. and Binder, J.R. (2006) Interrupting the "stream of consciousness": an fMRI investigation. NeuroImage, 29, 1185–1191. 16269249 10.1016/j.neuroimage.2005.09.030 PMC1634934

[dneu22701-bib-0071] Mckiernan, K.A. , Kaufman, J.N. , Kucera‐Thompson, J. and Binder, J.R. (2003) A parametric manipulation of factors affecting task‐induced deactivation in functional neuroimaging. Journal of Cognitive Neuroscience, 15, 394–408. 12729491 10.1162/089892903321593117

[dneu22701-bib-0072] Merabet, L. , Swisher, J.D. , McMains, S.A. , Halko, M.A. , Amedi, A. , Pascual‐Leone, A. *et al.* (2007) Combined activation and deactivation of visual cortex during tactile sensory processing. Journal of Neurophysiology, 97, 1633–1641. 17135476 10.1152/jn.00806.2006

[dneu22701-bib-0073] Minzenberg, M.J. , Yoon, J.H. and Carter, C.S. (2011) Modafinil modulation of the default mode network. Psychopharmacology, 215, 23–31. 21153806 10.1007/s00213-010-2111-5 PMC3072511

[dneu22701-bib-0074] Moraschi, M. , DiNuzzo, M. and Giove, F. (2012) On the origin of sustained negative BOLD response. Journal of Neurophysiology, 108, 2339–2342. 22723671 10.1152/jn.01199.2011

[dneu22701-bib-0075] Morita, T. , Saito, D.N. , Ban, M. , Shimada, K. , Okamoto, Y. , Kosaka, H. *et al.* (2018) Self‐face recognition begins to share active region in right inferior parietal lobule with proprioceptive illusion during adolescence. Cerebral Cortex, 28, 1532–1548. 29420750 10.1093/cercor/bhy027 PMC6093481

[dneu22701-bib-0076] Morosan, P. , Rademacher, J. , Schleicher, A. , Amunts, K. , Schormann, T. and Zilles, K. (2001) Human primary auditory cortex: cytoarchitectonic subdivisions and mapping into a spatial reference system. NeuroImage, 13, 684–701. 11305897 10.1006/nimg.2000.0715

[dneu22701-bib-0077] Mullinger, K.J. , Mayhew, S.D. , Bagshaw, A.P. , Bowtell, R. and Francis, S.T. (2014) Evidence that the negative BOLD response is neuronal in origin: A simultaneous EEG–BOLD–CBF study in humans. NeuroImage, 94, 263–274. 24632092 10.1016/j.neuroimage.2014.02.029

[dneu22701-bib-0078] Naccarato, M. , Calautti, C. , Jones, P.S. , Day, D.J. , Carpenter, T.A. and Baron, J.C. (2006) Does healthy aging affect the hemispheric activation balance during paced index‐to‐thumb opposition task? An fMRI study. NeuroImage, 32, 1250–1256. 16806984 10.1016/j.neuroimage.2006.05.003

[dneu22701-bib-0079] Naito, E. and Hirose, S. (2014) Efficient foot motor control by Neymar's brain. Frontiers in Human Neuroscience, 8, 594. 25136312 10.3389/fnhum.2014.00594 PMC4118031

[dneu22701-bib-0080] Naito, E. , Kinomura, S. , Geyer, S. , Kawashima, R. , Roland, P.E. and Zilles, K. (2000) Fast reaction to different sensory modalities activates common fields in the motor areas, but the anterior cingulate cortex is involved in the speed of reaction. Journal of Neurophysiology, 83, 1701–1709. 10712490 10.1152/jn.2000.83.3.1701

[dneu22701-bib-0081] Naito, E. , Morita, T. and Asada, M. (2016) Immature cerebro‐cerebellar interaction for timing motor control in children 22nd Annual Meeting of the Organization for Human Brain Mapping, Geneva, Switzerland, Poster number 2000.

[dneu22701-bib-0082] Naito, E. , Morita, T. , Saito, D.N. , Ban, M. , Shimada, K. , Okamoto, Y. *et al.* (2017) Development of right‐hemispheric dominance of inferior parietal lobule in proprioceptive illusion task. Cerebral Cortex, 27, 5385–5397. 28968653 10.1093/cercor/bhx223 PMC5939204

[dneu22701-bib-0083] Naito, E. , Nakashima, T. , Kito, T. , Aramaki, Y. , Okada, T. and Sadato, N. (2007) Human limb‐specific and non‐limb‐specific brain representations during kinesthetic illusory movements of the upper and lower extremities. European Journal of Neuroscience, 25, 3476–3487. 17553017 10.1111/j.1460-9568.2007.05587.x

[dneu22701-bib-0084] Nebel, M.B. , Joel, S.E. , Muschelli, J. , Barber, A.D. , Caffo, B.S. , Pekar, J.J. *et al.* (2014) Disruption of functional organization within the primary motor cortex in children with autism. Human Brain Mapping, 35, 567–580. 23118015 10.1002/hbm.22188 PMC3864146

[dneu22701-bib-0085] Newton, J.M. , Sunderland, A. and Gowland, P.A. (2005) fMRI signal decreases in ipsilateral primary motor cortex during unilateral hand movements are related to duration and side of movement. NeuroImage, 24, 1080–1087. 15670685 10.1016/j.neuroimage.2004.10.003

[dneu22701-bib-0086] Northoff, G. , Heinzel, A. , De Greck, M. , Bermpohl, F. , Dobrowolny, H. and Panksepp, J. (2006) Self‐referential processing in our brain‐a meta‐analysis of imaging studies on the self. NeuroImage, 31, 440–457. 16466680 10.1016/j.neuroimage.2005.12.002

[dneu22701-bib-0087] Ochsner, K.N. , Beer, J.S. , Robertson, E.R. , Cooper, J.C. , Gabrieli, J.D. , Kihsltrom, J.F. *et al.* (2005) The neural correlates of direct and reflected self‐knowledge. NeuroImage, 28, 797–814. 16290016 10.1016/j.neuroimage.2005.06.069

[dneu22701-bib-0088] Oldfield, R.C. (1971) The assessment and analysis of handedness: the Edinburgh inventory. Neuropsychologia, 9, 97–113. 5146491 10.1016/0028-3932(71)90067-4

[dneu22701-bib-0089] O'Reilly, J.X. , Beckmann, C.F. , Tomassini, V. , Ramnani, N. and Johansen‐Berg, H. (2010) Distinct and overlapping functional zones in the cerebellum defined by resting state functional connectivity. Cerebral Cortex, 20, 953–965. 19684249 10.1093/cercor/bhp157 PMC2837094

[dneu22701-bib-0090] Palmer, L.M. , Schulz, J.M. , Murphy, S.C. , Ledergerber, D. , Murayama, M. and Larkum, M.E. (2012) The cellular basis of GABA(B)‐mediated interhemispheric inhibition. Science, 335, 989–993. 22363012 10.1126/science.1217276

[dneu22701-bib-0091] Pasley, B.N. , Inglis, B.A. and Freeman, R.D. (2007) Analysis of oxygen metabolism implies a neural origin for the negative BOLD response in human visual cortex. NeuroImage, 36, 269–276. 17113313 10.1016/j.neuroimage.2006.09.015 PMC2001204

[dneu22701-bib-0092] Pecenka, N. and Keller, P.E. (2011) The role of temporal prediction abilities in interpersonal sensorimotor synchronization. Experimental Brain Research, 211, 505–515. 21424257 10.1007/s00221-011-2616-0

[dneu22701-bib-0093] Pfeifer, J.H. , Lieberman, M.D. and Dapretto, M. (2007) ‘I know you are but what am I ?!’: neural bases of self‐ and social knowledge retrieval in children and adults. Journal of Cognitive Neuroscience, 19, 1323–1337. 17651006 10.1162/jocn.2007.19.8.1323 PMC3407805

[dneu22701-bib-0094] Picard, N. , Matsuzaka, Y. and Strick, P.L. (2013) Extended practice of a motor skill is associated with reduced metabolic activity in M1. Nature Neuroscience, 16, 1340–1347. 23912947 10.1038/nn.3477 PMC3757119

[dneu22701-bib-0095] Power, J.D. , Barnes, K.A. , Snyder, A.Z. , Schlaggar, B.L. and Petersen, S.E. (2012) Spurious but systematic correlations in functional connectivity MRI networks arise from subject motion. NeuroImage, 59, 2142–2154. 22019881 10.1016/j.neuroimage.2011.10.018 PMC3254728

[dneu22701-bib-0096] Price, C.J. and Friston, K.J. (1997) Cognitive conjunction: a new approach to brain activation experiments. NeuroImage, 5, 261–270. 9345555 10.1006/nimg.1997.0269

[dneu22701-bib-0097] Riecker, A. , Gröschel, K. , Ackermann, H. , Steinbrink, C. , Witte, O. and Kastrup, A. (2006) Functional significance of age‐related differences in motor activation patterns. NeuroImage, 32, 1345–1354. 16798017 10.1016/j.neuroimage.2006.05.021

[dneu22701-bib-0098] Raichle, M.E. , MacLeod, A.M. , Snyder, A.Z. , Powers, W.J. , Gusnard, D.A. and Shulman, G.L. (2001) A default mode of brain function. Proceedings of the National Academy of Sciences, 98, 676–682. 10.1073/pnas.98.2.676 PMC14647 11209064

[dneu22701-bib-0099] Raichle, M.E. and Snyder, A.Z. (2007) A default mode of brain function: a brief history of an evolving idea. NeuroImage, 37, 1083–1090. 17719799 10.1016/j.neuroimage.2007.02.041

[dneu22701-bib-0100] Ruff, C.C. , Bestmann, S. , Blankenburg, F. , Bjoertomt, O. , Josephs, O. , Weiskopf, N. *et al.* (2008) Distinct causal influences of parietal versus frontal areas on human visual cortex: evidence from concurrent TMS‐fMRI. Cerebral Cortex, 18, 817–827. 17652468 10.1093/cercor/bhm128 PMC2601025

[dneu22701-bib-0101] Ruff, C.C. , Blankenburg, F. , Bjoertomt, O. , Bestmann, S. , Freeman, E. , Haynes, J.D. *et al.* (2006) Concurrent TMS‐fMRI and psychophysics reveal frontal influences on human retinotopic visual cortex. Current Biology, 16, 1479–1488. 16890523 10.1016/j.cub.2006.06.057

[dneu22701-bib-0102] Ruff, C.C. , Blankenburg, F. , Bjoertomt, O. , Bestmann, S. , Weiskopf, N. and Driver, J. (2009) Hemispheric differences in frontal and parietal influences on human occipital cortex: direct confirmation with concurrent TMS‐fMRI. Journal of Cognitive Neuroscience, 21, 1146–1161. 18752395 10.1162/jocn.2009.21097 PMC2667814

[dneu22701-bib-0103] Sadato, N. , Pascual‐Leone, A. , Grafman, J. , Deiber, M.P. , Ibanez, V. and Hallett, M. (1998) Neural networks for Braille reading by the blind. Brain: A Journal of Neurology, 121, 1213–1229. 9679774 10.1093/brain/121.7.1213

[dneu22701-bib-0104] Sadato, N. , Pascual‐Leone, A. , Grafman, J. , Ibañez, V. , Deiber, M.P. , Dold, G. *et al.* (1996) Activation of the primary visual cortex by Braille reading in blind subjects. Nature, 380, 526–528. 8606771 10.1038/380526a0

[dneu22701-bib-0105] Sang, L. , Qin, W. , Liu, Y. , Han, W. , Zhang, Y. , Jiang, T. *et al.* (2012) Resting‐state functional connectivity of the vermal and hemispheric subregions of the cerebellum with both the cerebral cortical networks and subcortical structures. NeuroImage, 61, 1213–1225. 22525876 10.1016/j.neuroimage.2012.04.011

[dneu22701-bib-0106] Schäfer, K. , Blankenburg, F. , Kupers, R. , Grüner, J.M. , Law, I. , Lauritzen, M. *et al.* (2012) Negative BOLD signal changes in ipsilateral primary somatosensory cortex are associated with perfusion decreases and behavioral evidence for functional inhibition. NeuroImage, 59, 3119–3127. 22155327 10.1016/j.neuroimage.2011.11.085

[dneu22701-bib-0107] Scheperjans, F. , Palomero‐Gallagher, N. , Grefkes, C. , Schleicher, A. and Zilles, K. (2005) Transmitter receptors reveal segregation of cortical areas in the human superior parietal cortex: relations to visual and somatosensory regions. NeuroImage, 28, 362–379. 16054841 10.1016/j.neuroimage.2005.06.028

[dneu22701-bib-0108] Schneider, C. , Devanne, H. , Lavoie, B.A. and Capaday, C. (2002) Neural mechanisms involved in the functional linking of motor cortical points. Experimental Brain Research, 146, 86–94. 12192582 10.1007/s00221-002-1137-2

[dneu22701-bib-0109] Schneider, D.M. , Sundararajan, J. and Mooney, R. (2018) A cortical filter that learns to suppress the acoustic consequences of movement. Nature, 561, 391–395. 30209396 10.1038/s41586-018-0520-5 PMC6203933

[dneu22701-bib-0110] Schridde, U. , Khubchandani, M. , Motelow, J.E. , Sanganahalli, B.G. , Hyder, F. and Blumenfeld, H. (2008) Negative BOLD with large increases in neuronal activity. Cerebral Cortex, 18, 1814–1827. 18063563 10.1093/cercor/bhm208 PMC2790390

[dneu22701-bib-0111] Sebastian, C. , Burnett, S. and Blakemore, S.J. (2008) Development of the self‐concept during adolescence. Trends in Cognitive Sciences, 12, 441–446. 18805040 10.1016/j.tics.2008.07.008

[dneu22701-bib-0112] Shih, Y.Y.I. , Chen, C.C.V. , Shyu, B.C. , Lin, Z.J. , Chiang, Y.C. , Jaw, F.S. *et al.* (2009) A new scenario for negative functional magnetic resonance imaging signals: endogenous neurotransmission. Journal of Neuroscience, 29, 3036–3044. 19279240 10.1523/JNEUROSCI.3447-08.2009 PMC6666445

[dneu22701-bib-0113] Shmuel, A. , Augath, M. , Oeltermann, A. and Logothetis, N.K. (2006) Negative functional MRI response correlates with decreases in neuronal activity in monkey visual area V1. Nature Neuroscience, 9, 569–577. 16547508 10.1038/nn1675

[dneu22701-bib-0114] Shmuel, A. , Yacoub, E. , Pfeuffer, J. , Van de Moortele, P.F. , Adriany, G. , Hu, X. *et al.* (2002) Sustained negative BOLD, blood flow and oxygen consumption response and its coupling to the positive response in the human brain. Neuron, 36, 1195–1210. 12495632 10.1016/s0896-6273(02)01061-9

[dneu22701-bib-0115] Shulman, G.L. , Fiez, J.A. , Corbetta, M. , Buckner, R.L. , Miezin, F.M. , Raichle, M.E. *et al.* (1997) Common blood flow changes across visual tasks: II. Decreases in cerebral cortex. Journal of Cognitive Neuroscience, 9, 648–663. 23965122 10.1162/jocn.1997.9.5.648

[dneu22701-bib-0116] Siegel, J.S. , Power, J.D. , Dubis, J.W. , Vogel, A.C. , Church, J.A. , Schlaggar, B.L. *et al.* (2014) Statistical improvements in functional magnetic resonance imaging analyses produced by censoring high‐motion data points. Human Brain Mapping, 35, 1981–1996. 23861343 10.1002/hbm.22307 PMC3895106

[dneu22701-bib-0117] Smith, A.T. , Williams, A.L. and Singh, K.D. (2004) Negative BOLD in the visual cortex: evidence against blood stealing. Human Brain Mapping, 21, 213–220. 15038003 10.1002/hbm.20017 PMC6871689

[dneu22701-bib-0118] Sten, S. , Lundengård, K. , Witt, S.T. , Cedersund, G. , Elinder, F. and Engström, M. (2017) Ne ural inhibition can explain negative BOLD responses: A mechanistic modelling and fMRI study. NeuroImage, 158, 219–231. 28687518 10.1016/j.neuroimage.2017.07.002

[dneu22701-bib-0119] Stepniewska, I. , Preuss, T.M. and Kaas, J.H. (1993) Architectonics, somatotopic organization, and ipsilateral cortical connections of the primary motor area (M1) of owl monkeys. Journal of Comparative Neurology, 330, 238–271. 7684050 10.1002/cne.903300207

[dneu22701-bib-0120] Takahashi, T. , Shirane, R. , Sato, S. and Yoshimoto, T. (1999) Developmental changes of cerebral blood flow and oxygen metabolism in children. American Journal of Neuroradiology, 20, 917–922. 10369366 PMC7056161

[dneu22701-bib-0121] Talelli, P. , Ewas, A. , Waddingham, W. , Rothwell, J.C. and Ward, N.S. (2008) Neural correlates of age‐related changes in cortical neurophysiology. NeuroImage, 40, 1772–1781. 18329904 10.1016/j.neuroimage.2008.01.039 PMC3715371

[dneu22701-bib-0122] Thomason, M.E. , Chang, C.E. , Glover, G.H. , Gabrieli, J.D. , Greicius, M.D. and Gotlib, I.H. (2008) Default‐mode function and task‐induced deactivation have overlapping brain substrates in children. NeuroImage, 41, 1493–1503. 18482851 10.1016/j.neuroimage.2008.03.029 PMC2735193

[dneu22701-bib-0123] Turesky, T.K. , Olulade, O.A. , Luetje, M.M. and Eden, G.F. (2018) An fMRI study of finger tapping in children and adults. Human Brain Mapping, 39, 3203–3215. Available at: 10.1002/hbm.24070. 29611256 PMC6052794

[dneu22701-bib-0124] Vogeley, K. and Fink, G.R. (2003) Neural correlates of the first‐person‐perspective. Trends in Cognitive Sciences, 7, 38–42. 12517357 10.1016/s1364-6613(02)00003-7

[dneu22701-bib-0125] Wade, A.R. and Rowland, J. (2010) Early suppressive mechanisms and the negative blood oxygenation level‐dependent response in human visual cortex. Journal of Neuroscience, 30, 5008–5019. 20371821 10.1523/JNEUROSCI.6260-09.2010 PMC3523120

[dneu22701-bib-0126] Ward, N.S. , Swayne, O.B. and Newton, J.M. (2008) Age‐dependent changes in the neural correlates of force modulation: an fMRI study. Neurobiology of Aging, 29, 1434–1446. 17566608 10.1016/j.neurobiolaging.2007.04.017 PMC2568861

[dneu22701-bib-0127] Weisser, V. , Stilla, R. , Peltier, S. , Hu, X. and Sathian, K. (2005) Short‐term visual deprivation alters neural processing of tactile form. Experimental Brain Research, 166, 572–582. 16086141 10.1007/s00221-005-2397-4

[dneu22701-bib-0128] Witt, S.T. , Laird, A.R. and Meyerand, M.E. (2008) Functional neuroimaging correlates of finger‐tapping task variations: an ALE meta‐analysis. NeuroImage, 42, 343–356. 18511305 10.1016/j.neuroimage.2008.04.025 PMC2592684

[dneu22701-bib-0129] Worsley, K.J. and Friston, K.J. (1995) Analysis of fMRI time‐series revisited–again. NeuroImage, 2, 173–181. 9343600 10.1006/nimg.1995.1023

