# Supplementary material for: Developmental Changes in Task‐Induced Brain Deactivation in Humans Revealed by a Motor Task
Source: Dev Neurobiol. 2019 Jun 10;79(6):536–58. doi: 10.1002/dneu.22701 (PMC6771882; doi:10.1002/dneu.22701)
Supplement: Supplementary file 1 [file DNEU-79-536-s001.pdf]

Supplementary Table 1 Brain deactivations in each group

Adults

Clusters	Size	x	y	z	T	Anatomical identification
Bilateral OTPF cluster	30698	52	-4	-14	8.93	Superior temporal gyrus
		24	-26	60	8.84	Postcentral gyrus
		42	-28	52	8.76	Area 3b
		34	-52	66	8.58	Area 7PC
		58	2	-8	8.33	Area TE3
		52	10	-16	7.92	Temporal ploe
		-30	-40	52	7.75	Area 2
		-18	-52	68	7.41	Area 5L
		52	-12	6	7.35	Area TE1.0
		-28	-50	70	7.10	Superior parietal lobule
		34	-18	48	7.06	Precentral gyrus
		46	-14	34	7.05	Area 4p
		-26	-64	60	7.01	Area 7A
		26	-6	66	6.82	Superior frontal gyrus
		54	-62	-10	6.72	Inferior temporal gyrus
		22	-62	64	6.70	Area 7A
		62	-34	0	6.67	Middle temporal gyrus
		14	-82	44	6.60	Area 7P
		-8	-34	50	6.51	Area 5M
		-8	-32	38	6.45	MCC
		8	-18	78	6.31	Posterior-medial frontal
		38	-42	66	6.16	Area 1
		14	-30	54	6.09	Paracentral lobule
		8	-56	12	6.08	Calcarine gyrus
		30	-24	-24	6.01	Parahippocampal gyrus
		-34	-86	32	6.00	Area PGp
		-44	-66	18	5.89	Middle temporal gyrus
		54	-2	16	5.86	Rolandic Operculum
		46	-26	10	5.83	Area TE1.1
		42	-12	14	5.80	Area OP3
		26	-82	34	5.80	Superior occipital gyrus
		46	-76	22	5.76	Area PGp
		-8	-34	78	5.75	Area 4a
		32	-72	38	5.67	Middle occipital gyrus
		44	-62	32	5.64	Angular gyrus
		-20	-86	44	5.64	area hOC4d [V3A]
		20	-62	20	5.60	Cuneus
		2	-50	46	5.60	Precuneus
		-28	-72	32	5.59	Middle occipital gyrus
		42	-26	36	5.56	Area PFt
		-28	-56	60	5.52	Area 7PC
		-62	-52	-12	5.33	Inferior temporal gyrus
		-10	-44	6	5.33	Calcarine gyrus
		-44	-82	4	5.25	Area hOc4la
Bilateral MPFC cluster	3769	24	58	4	6.22	Area Fp1
		-14	60	0	5.74	Area Fp1
		-2	10	-10	5.26	Area 25
		-8	40	-18	5.24	Area Fo1
		6	46	-16	5.08	Area Fp2
		-6	60	-2	4.91	Area Fp2
		-22	40	-10	4.82	Middle orbital gyrus
		-12	16	-2	4.79	Caudate Nucleus
		-42	28	-10	4.77	IFG (Orbitalis)
		12	24	-14	4.71	Area Fo2
		8	36	-20	4.55	Area Fo1
		-10	36	-10	4.54	Area s32
		-38	22	-22	4.53	Temporal pole
		26	22	-24	4.41	Area Fo3
		-26	32	-16	4.37	Area Fo3
Left temporal cluster	2527	-50	-24	2	8.35	Superior temporal gyrus
		-64	-20	2	7.14	Area TE3
		-28	0	-26	6.97	Amygdala
		-38	-20	4	5.61	Area lg2

Adolescents

Clusters	Size	x	y	z	T	Anatomical identification
Bilateral OTPF cluster	25486	24	-54	70	9.68	Area 7PC
		40	-30	62	9.62	Area 4a
		38	-26	50	9.57	Area 4p
		10	-82	50	9.48	Area 7P
		22	-48	62	9.23	Area 5L
		-18	-44	60	8.23	Area 5L
		12	-42	64	8.08	Precuneus
		24	-74	38	8.04	Superior occipital gyrus
		-8	-62	62	8.00	Area 7A
		62	-10	-2	7.82	Area TE3
		16	-62	66	7.80	Area 7A
		-24	-40	64	7.52	Area 3b
		-20	-82	42	7.49	Superior occipital gyrus
		46	-18	54	7.36	Postcentral gyrus
		46	-74	18	7.16	Area PGp
		30	-8	58	7.13	Superior frontal gyrus
		22	-36	58	6.99	Area 3b
		12	-76	40	6.95	Cuneus
		34	-26	70	6.93	Precentral gyrus
		6	-44	58	6.92	Area 5M
		-28	-56	62	6.90	Area 7PC
		36	-52	68	6.81	Superior parietal lobule
		-6	-50	72	6.79	Area 5M
		46	-62	-4	6.54	Inferior temporal gyrus
		52	-26	60	6.49	Area 1
		32	-64	36	6.43	Middle occipital gyrus
		12	-26	52	6.32	Posterior-medial frontal
		-2	-30	68	6.29	Area 4a
		58	-42	12	6.23	Superior temporal gyrus
		-46	-68	-6	6.02	Inferior temporal gyrus
		-38	-80	10	5.96	Area hOc4la
		-40	-32	38	5.71	Area PFt
		-4	-72	58	5.70	Area 7P
		-18	-86	34	5.67	Area hOc4d [V3A]
		50	-54	6	5.60	Middle temporal gyrus
		48	-28	14	5.58	Area OP1
		26	-40	48	5.46	Area 2
		-24	-66	44	5.45	Inferior parietal lobule
		42	-14	6	5.45	Area lg2
		-44	-84	14	5.40	Area hOc4lp
		-36	-46	68	5.39	Superior parietal lobule
		50	-70	-2	5.34	Area hOc4la
		14	-34	58	5.27	Paracentral lobule
		52	-14	12	5.26	Rolandic operculum
Left MPFC cluster	305	-26	60	-2	6.41	Area Fp1
		-36	60	4	5.17	Middle frontal gyrus
Left MPFC cluster	200	-18	10	-20	6.59	Area Fo2
		-10	10	-14	3.33	Olfactory cortex
Left temporal cluster	1639	-60	-16	2	6.22	Area TE3
		-48	-28	4	5.91	Superior temporal gyrus
		-54	-42	0	5.09	Middel temporal gyrus
		-64	-8	16	4.01	Area OP4

Children

Clusters	Size	x	y	z	T	Anatomical identification
Right fronto-parietal cluster	3894	36	-20	68	6.62	Precentral gyrus
		32	-42	62	6.43	Area 2
		40	-40	66	5.78	Area 1
		40	-22	38	5.66	Area 3b
		34	-32	50	5.56	Area 4p
		26	-58	70	5.26	Area 7A
		10	-72	60	5.11	Area 7P
		8	-54	68	4.68	Area 5L
		18	-68	50	4.55	Superior parietal lobule
		34	-50	68	4.55	Area 7PC
		20	-36	72	4.18	Area 4a
		20	-10	64	4.04	Superior frontal gyrus
		6	-46	72	3.87	Precuneus
		2	-48	62	3.37	Area 5M
		14	-42	76	3.17	Postcentral gyrus
Right temporal cluster	1661	38	-24	8	6.56	Area TE1.1
		52	-16	2	6.12	Area TE1.0
		56	-2	-2	5.79	Area TE1.2
		52	-10	-6	5.67	Superior temporal gyrus
		34	-12	0	5.34	Putamen
		46	-16	10	5.25	Heschls gyrus
		22	-30	10	4.97	Thal: Parietal
		54	6	-10	4.35	Temporal pole
		52	-20	18	4.18	Area OP1
		66	-10	10	4.17	Area OP4
		16	-22	18	4.13	Thal: temporal
		38	-14	22	3.73	Insula
		66	-14	2	3.59	Area TE3
		32	-12	-14	3.58	Hippocampus
Left parietal cluster	1295	-30	-46	64	6.48	Area 5L
		-46	-32	48	5.08	Area 2
		-32	-46	56	5.07	Area 7PC
		-12	-78	52	4.73	Area 7P
		-10	-60	66	4.67	Area 7A
		-10	-66	48	4.63	Precuneus
		-38	-50	48	3.57	Area hIP2
		-42	-44	56	3.56	Inferior parietal lobule
		-44	-32	40	3.36	Area PFt
		-22	-72	50	3.34	Superior parietal lobule
Right paracentral cluster	162	8	-34	50	5.16	Area 5Ci
		6	-22	54	3.90	Area 4a
Left temporal cluster	690	-42	-22	6	5.63	Area TE1.1
		-46	-14	-2	4.62	Superior temporal gyrus
		-66	-28	12	4.59	Area TE3

		-26	-10	-18	5.39	Hippocampus			-44	-38	14	3.13	Area PFcm							
		-52	6	-12	5.19	Temporal ploe														
		-52	-32	2	5.16	Middel temporal gyrus	Left hippocampal cluster	159	-28	-10	-24	7.14	Area CA1(Hippocampus)							
		-32	-26	14	5.09	Area lg1			-44	-12	-28	4.68	Inferior temporal gyrus							
		-50	14	-32	3.61	Medial temporal pole			-22	0	-32	4.53	Entorhinal cortex							
Left SM1 cluster	414	-46	-10	36	5.90	Postcentral gyrus														
Left superior frontal cluster	330	-22	46	34	5.35	Middle frontal gyrus														
		-20	36	32	4.25	Superior frontal gyrus														
		-14	40	26	3.26	Superior mediall gyrus														
Left IFG cluster	210	-38	22	18	4.71	IFG (triangularis)														
Left occipital cluster	172	-18	-92	-2	3.75	Middle occipital gyrus														
		-20	-80	-2	3.56	Area hOc4v (V4v)														
		-18	-98	-12	3.35	Area hOc3v (V3v)														
		-30	-70	-8	3.32	Area FG1														
Right superior frontal cluster	162	18	32	34	4.10	Superior frontal gyrus														
		26	30	46	4.07	Middle frontal gyrus														
Left posterior cerebellar cluster	521	-16	-74	-34	7.67	Lobule VIIa CrusI	Left posterior cerebellar cluster	415	-16	-78	-40	5.43	Lobule VIIa CrusII	Left posterior cerebellar cluster	204	-10	-76	-28	4.36	Lobule VI (Hem)
		-12	-82	-38	5.39	Lobule VIIa CrusII			-16	-82	-32	4.55	Lobule VIIa CrusI			-20	-76	-36	4.09	Lobule VIIa CrusII
		-8	-76	-34	4.98	Crus II										-10	-74	-42	3.67	Lobule VIIb (Hem)
		-28	-58	-42	3.83	Cerebellum (VIII)										-14	-68	-32	2.90	Cerebellum (Crus 1)
Left cerebellar cluster	183	-44	-56	-44	4.39	Lobule VIIa CrusI	Left cerebellar cluster	202	-26	-46	-32	4.71	Lobule VI (Hem)							
									-18	-50	-24	4.48	Lobule V (Hem)							
Bilateral inferior cerebellar clust	586	-10	-50	-50	5.01	Lobule IX (Hem)														
		4	-50	-50	4.90	Lobule IX (Hem)														
		-12	-60	-44	4.42	Cerebelum (VIII)														
		-8	-44	-44	4.33	Cerebelum (IX)														
		-18	-54	-50	3.94	Lobule VIIIb (Hem)														
		-24	-48	-48	3.66	Lobule VIIIa (Hem)														
Right posterior cerebellar cluster	174	12	-82	-36	4.43	Lobule VIIa CrusII	Right cerebellar cluster	166	24	-76	-40	4.12	Lobule VIIa CrusII							
		10	-78	-28	3.54	Lobule VIIa CrusI			24	-72	-50	3.92	Lobule VIIb (Hem)							
Right thalamus cluster	144	22	-20	8	4.65	Thal: Somatosensory														
		14	-28	4	4.10	Thal: Temporal														
		16	-28	-4	4.09	Thal: Parietal														

See footnote in Table 1. In each cluster, we reported peaks that were more than 8 mm apart from each other in order of larger T-values. We reported top 44 peaks in the bilateral OTPF clusters of the AD and the ADO groups, and top 15 peaks in the MPFC cluster for the AD group. Abbreviations: OTPF, occipito-temporo-parieto-frontal; MPFC, medial prefrontal cortex; SM1, primary sensorimotor cortex; IFG, inferior frontal gyrus; MCC, middle cingulate cortex
