# Supplementary material for: Developmental Changes in Task‐Induced Brain Deactivation in Humans Revealed by a Motor Task
Source: Dev Neurobiol. 2019 Jun 10;79(6):536–58. doi: 10.1002/dneu.22701 (PMC6771882; doi:10.1002/dneu.22701)
Supplement: Supplementary file 2 [file DNEU-79-536-s002.pdf]

Supplementary Table 2 Brain activations in each group

	Adults						Adolescents						Children					
Clusters	Size	x	y	z	T	Anatomical identification	Size	x	y	z	T	Anatomical identification	Size	x	y	z	T	Anatomical identification
Motor network																		
Left SM1 cluster	166	-32	-26	54	5.29	Area 4p	494	-34	-24	54	5.77	Area 4p	1231	-32	-22	54	11.06	Precentral gyrus
		-34	-12	50	3.93	Precentral gyrus		-42	-14	64	5.70	Precentral gyrus		-48	-16	44	4.79	Area 3b
								-40	-16	52	5.02	Area 4a		-52	-12	52	4.68	Postcentral gyrus
								-34	-32	72	3.60	Postcentral gyrus						
Right cerebellar cluster	1774	14	-54	-20	9.48	Lobule VI (Hem)	1403	12	-56	-14	10.99	Lobule VI (Hem)	1843	16	-50	-22	11.32	Lobule V (Hem)
		8	-58	-12	7.48	Lobule V (Hem)		8	-58	-6	7.16	Lobule V (Hem)		4	-66	-16	8.10	Lobule VI (Hem)
		4	-66	-24	5.43	Lobule VI (Vermis)		2	-60	0	4.48	Vermis		6	-40	-18	3.61	Lobule I IV (Hem)
		42	-62	-30	3.84	Lobule VIIa (Crus I)		0	-48	-4	4.25	Lobule I IV (Hem)		30	-42	-16	3.49	Area FG3
														20	-70	-2	3.21	Lingual gyrus
														2	-74	-10	2.99	Lobule VI (Verimis)
Left subcortical cluster	142	-16	-20	-2	6.88	Thal: Motor	158	-14	-22	8	7.56	Thal: Prefrontal	370	-18	-22	-2	5.57	Thal: Parietal
								-18	-18	-2	4.55	Thal: Motor		-22	-8	-2	5.21	Pallidum
														-30	-16	2	4.85	Putamen
														-8	-20	-2	3.90	Thal: Prefrontal
Outside of Motor network																		
Left																		
Insular cluster	412	-44	0	2	8.20	Insula	343	-42	-4	10	6.19	Rolandic Operculum	215	-50	2	6	5.66	Rolandic Operculum
		-50	4	6	4.89	IFG (Opercularis)		-44	6	-2	3.55	Insula		-46	8	0	3.56	Area 44
		-52	10	6	4.30	Area 44												
		-38	6	2	3.92	Insula												
Cerebellar cluster	156	-24	-64	-22	6.78	Lovule VI (Hem)												
Right																		
IFG/insular cluster	844	56	14	12	7.32	Area 44	512	44	8	-2	6.99	Insula	355	40	8	6	5.93	Insula
		42	4	-4	5.77	Insula		44	10	8	5.98	IFG (Opercularis)		38	18	12	4.81	IFG (Opercularis)
		36	22	10	4.30	IFG (Triangularis)		52	10	10	4.87	Area 44		50	6	8	4.41	Rolandic Operculum
														48	16	4	3.41	Area 44
													184	42	50	2	4.62	Middle frontal gyrus
														50	42	2	3.57	IFG (Triangularis)
Inferior parietal cluster	267	48	-46	48	4.87	Area hIP2	126	64	-28	46	4.85	SupraMarginal gyrus						
		52	-42	42	4.17	Area PFm		64	-36	40	4.20	Area PF						
		54	-34	32	4.14	Area PFcm		66	-46	30	3.37	Area PFm						
		62	-30	48	4.10	SupraMarginal gyrus		62	-36	48	3.29	Inferior parietal lobule						
		38	-42	40	3.93	Area hIP1												
		60	-42	48	3.36	Inferior parietal lobule												
Medial prefrontal cortex																		
Left MPFC cluster													413	-2	60	24	4.71	Superior medial gyrus
														-8	58	6	4.23	Area Fp2
														-18	56	18	4.06	Superior frontal gyrus
														-18	60	10	3.92	Area Fp1
														0	52	12	2.97	ACC
Left MPFC cluster													224	-8	-6	66	5.85	Posterior medial frontal

Right MPFC cluster	141	16	50	36	4.37	Superior frontal gyrus
		10	58	34	4.05	Superior medial gyrus

See footnote in Table 1. Abbreviations: SM1, primary sensorimotor cortex; IFG, inferior frontal gyrus; MPFC, medial prefrontal cortex; ACC, anterior cingulate cortex
