# Supplementary material for: Developmental Changes in Task‐Induced Brain Deactivation in Humans Revealed by a Motor Task
Source: Dev Neurobiol. 2019 Jun 10;79(6):536–58. doi: 10.1002/dneu.22701 (PMC6771882; doi:10.1002/dneu.22701)
Supplement: Supplementary file 3 [file DNEU-79-536-s003.docx]

**Supplementary Material**

***Brain Regions in which Activity Correlated with Age***

We found that deactivation in the ipsilateral SM1 and left lateral occipital region was significantly advanced (Figure 3A), and activation in the contralateral SM1 was significantly smaller (Figure 5A), in the ADO group compared with the CH group. These results indicated that activity in these regions changed from childhood to adolescence. To confirm this hypothesis, we performed a correlation analysis. Using age (months) as a covariate, we identified brain regions with correlating activity in all 40 children and adolescents. For statistical inference, we used an FDR-corrected cluster-wise threshold of *p* < 0.05 in the entire brain space for a cluster image of voxels in which activity was correlated with age using an uncorrected voxel-wise threshold of *p* < 0.005.

We found regions in which activity was negatively correlated with age in the transition from childhood to adolescence (Supplementary Figure 1A). However, we found no regions in which activity was positively correlated with age. We confirmed that ipsilateral SM1 deactivation progressed with increasing age (Supplementary Figure 1B). Similarly, almost half of the child participants showed activation in the left lateral occipital cortex and its deactivation became robust in the majority of adolescent participants (Supplementary Figure 1C). In addition to these regions, we also found that activity was negatively correlated with age in the left SPL, bilateral PPC, and posterior occipital cortex (Supplementary Figure 1D-G). Thus, deactivation in these regions also gradually progressed during the childhood-to-adolescence transition, though the mean deactivation did not significantly differ between these two groups (CH vs. ADO, Figure 3A).

Finally, we confirmed that activity in the contralateral M1 gradually decreased from childhood to adolescence (Supplementary Figure 1H), consistent with the significant reduction in contralateral SM1 activation in the ADO group when compared with the CH group (Figure 5A).


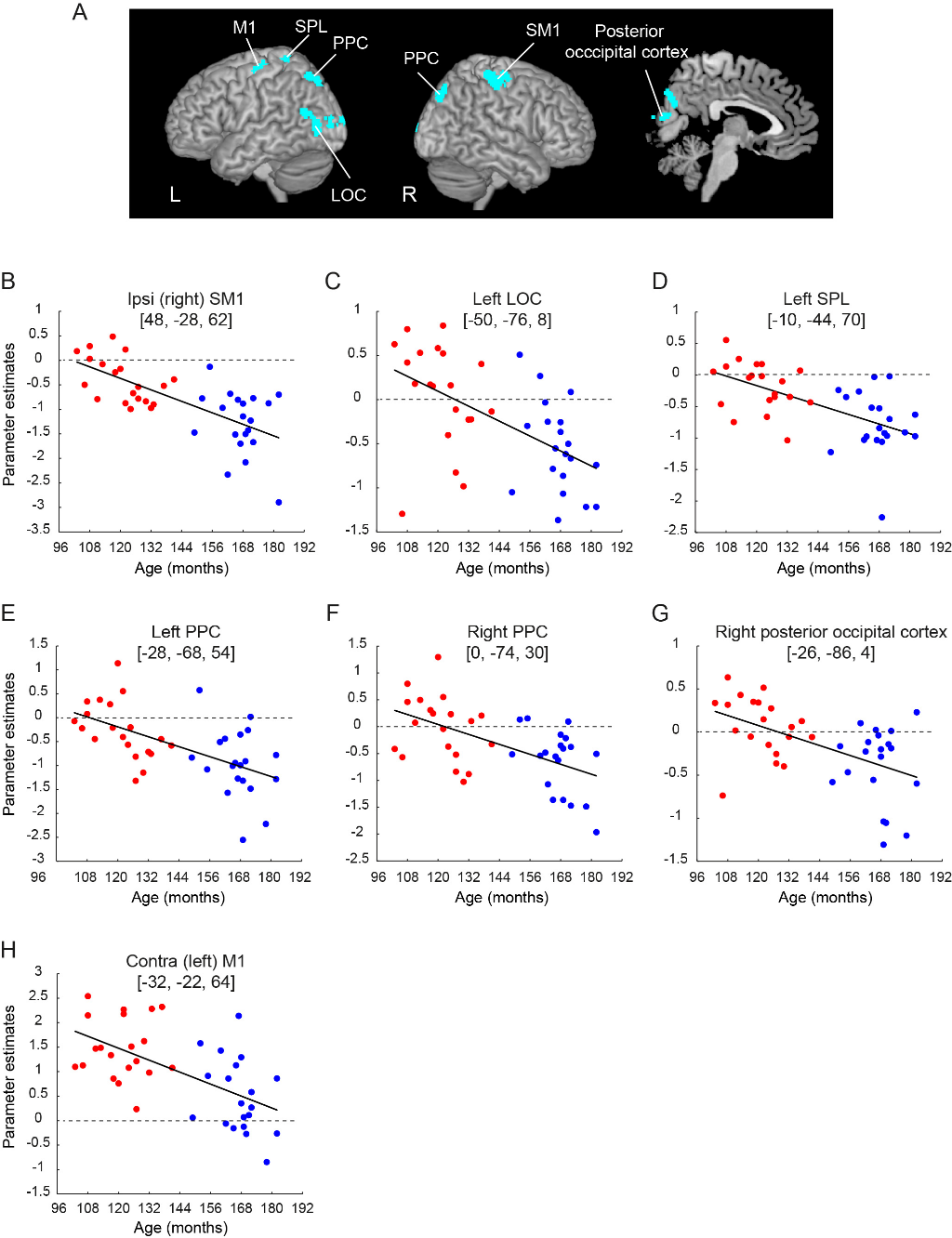
Supplementary Figure 1. The brain regions in which activity was negatively correlated with age during the transition from childhood to adolescence. (A) Deactivation (light blue) was rendered onto the left and right hemispheres, and a sagittal section (x = +4) of the MNI standard brain. (B-H) The correlation between age and brain activity (parameter estimate) in each region. Each dot represents an individual. Data obtained from the CH group are shown in red, and those of the ADO group are blue. The solid line in each panel represents a regression line fitted to the individual data (n = 40). Abbreviations: L, left; LOC, lateral occipital cortex; MNI, Montreal Neurological Institute; M1, primary motor cortex; PPC, posterior parietal cortex; R, right; SM1, primary sensorimotor cortex; SPL, superior parietal lobule
